# Metagenomic identification of disease-causing Salmonella enterica serovars and antimicrobial resistance genes from paediatric faecal samples

**DOI:** 10.1099/mgen.0.001547

**Published:** 2025-10-27

**Authors:** Agata H. Dziegiel, Vu Thuy Duong, Samuel J. Bloomfield, Nicholas R. Thomson, Duncan J. Maskell, John Wain, Nicol Janecko, Stephen Baker, Alison E. Mather

**Affiliations:** 1 Quadram Institute Bioscience, Norwich Research Park, Norwich, UK; 2 Centre for Microbial Interactions, Norwich Research Park, Norwich, UK; 3 Children’s Hospital 1, Ho Chi Minh City, Vietnam; 4 Wellcome Sanger Institute, Wellcome Genome Campus, Hinxton CB10 1SA, UK; 5 London School of Hygiene and Tropical Medicine, London, UK; 6 University of Melbourne, Melbourne, Australia; 7 University of East Anglia, Norwich Research Park, Norwich, UK; 8 Department of Medicine, University of Cambridge School of Clinical Medicine, Cambridge Biomedical Campus, Cambridge, UK

**Keywords:** antimicrobial resistance, enterocolitis, gut microbiome, metagenomics, nontyphoidal *Salmonella*, paediatric infections

## Abstract

**Background.** Nontyphoidal *Salmonella* (NTS) is a common cause of enterocolitis and a major cause of death in children in low- and middle-income countries (LMICs). High antimicrobial resistance (AMR) prevalence in LMICs reduces treatment options for individuals at risk of severe infections.

**Methods.** We investigated the use of metagenomics to identify NTS and associated AMR genes in 28 faecal metagenomes from children with culture-confirmed salmonellosis in Vietnam, using accompanying NTS genomes from isolated serovars (one per metagenome). Read-based and assembly-based methods were utilised for NTS and AMR detection. Case metagenomes were compared to healthy control metagenomes (*n*=21) with respect to the microbiome composition, NTS relative abundances, number of unique AMR genes and antimicrobial classes to which the genes confer resistance, including classes used in *Salmonella* treatment.

**Results.** Salmonellosis cases displayed significantly higher relative abundances of Enterobacteriaceae than controls. Bracken and Centrifuge analysis facilitated the identification of *Salmonella enterica* sequences in case metagenomes at varying relative abundances (0.00259–27.7 % of total reads), which were significantly higher than controls. MetaPhlAn4 did not detect *S. enterica* in any control metagenomes, though 12 case metagenomes were also negative. The isolated serovars were identified in 78.6% of the associated case metagenomes with Centrifuge, suggesting this method is the most sensitive; however, the isolated genome serovar was the most abundant in only six case metagenomes, and serovar sequences were also identified in control metagenomes. Alignment to a *Salmonella* reference database, followed by local assembly and realignment, predicted the isolated serovar as the most likely serovar present in 35.7% of metagenomes, whereas *Salmonella in silico* typing resource classification of the local assembly was concordant with the isolate genome in 28.6% of cases. Metagenome-assembled genomes produced using two tools following *de novo* assembly identified the isolated serovar in 17.8–21.4% of cases. The percentage of NTS AMR genes identified in each case metagenome ranged between 0.00 and 100%. There was no significant difference in the number of unique AMR genes or antimicrobial classes between cases and controls, indicating comparable resistomes between cohorts.

**Conclusions.** This study highlights the potential of metagenomics for NTS identification in faecal samples, although overlap in *S. enterica* relative abundance between cohorts calls for further work to identify a diagnostic cutoff. Reliable characterisation of the organism to the serovar and AMR genotype level is affected by the complexity of the microbiome, sequencing and analysis approaches. Increased sequencing depth, for example through improved host DNA depletion, may facilitate enhanced characterisation. Detection of multiple serovars within individual samples with the Centrifuge suggests inaccurate classification or the presence of multiple serovars, making characterisation difficult.

**Impact Statement**

Nontyphoidal *Salmonella* (NTS) is a common cause of diarrhoea. Paediatric infections are associated with higher rates of complications and severe disease, particularly in low- and middle-income countries like Vietnam. Clinical isolates of NTS in Vietnam often show high levels of antimicrobial resistance (AMR), which can limit treatment options in severe disease. Shotgun metagenomic sequencing allows examination of the whole microbial community within samples and could become the method of choice for diagnosis and epidemiologically relevant typing of infectious intestinal disease. It is important that the databases used to interpret these data are populated with DNA sequences representing different communities. The examination of the whole microbial community within stool samples from Vietnam is an important part of the process to potentiate direct identification and characterization of pathogens and microbiome patterns. This study assessed the use of metagenomics for the identification and characterization of NTS from faecal samples from children with confirmed salmonellosis (case metagenomes) and compared the results to healthy controls. The cases were marked with statistically significant differences in the microbiome compared to controls. The percentage of the sample classified as *Salmonella enterica* was also significantly higher in the case metagenomes compared to the controls. Characterisation of the serovar isolated from the case samples was possible in up to 78.6% of case metagenomes, but identification of low-abundance NTS serovar sequences in the control metagenomes may indicate low specificity of existing analysis approaches. NTS-associated AMR genes were identified in most case metagenomes, although many of these were likely carried by multiple organisms. There was no significant difference in the quantity of unique AMR genes or antimicrobial classes to which they confer resistance between the case and control metagenomes, indicating that although metagenomics can facilitate NTS identification in faecal samples, reliable characterisation for treatment guidance remains difficult.

## Data Summary

The authors confirm that all supporting data have been provided within the article or through supplementary data files.

## Introduction

*Salmonella enterica* are Gram-negative, facultative anaerobic bacilli in the *Enterobacteriaceae* family. Of the six subspecies described, *S. enterica* subspecies *enterica* is the most diverse, currently comprising over 1,500 serovars [1,2], many of which are clinically relevant. Contaminated food and water are the leading sources of nontyphoidal salmonellosis [3]. In comparison to the systemic disease associated with typhoidal *S. enterica*, nontyphoidal *Salmonella* (NTS) infections are generally characterised by mild to moderate enterocolitis [4]. However, paediatric NTS infections are associated with higher complication rates and subsequent invasive disease, particularly in low- and middle-income countries (LMICs), where diarrhoea is a leading cause of death in children [5].

Vietnam is an LMIC burdened with NTS infections in children [6]. Common clinically relevant serovars include *Salmonella* Typhimurium, *Salmonella* Weltevreden and *Salmonella* Stanley. *S*. Typhimurium, in particular, is often identified with high levels of antimicrobial resistance (AMR), resulting in nonsusceptibility to clinically important drug classes including beta-lactams, phenicols, fluoroquinolones and macrolides [7]. High antimicrobial consumption in Vietnam may have led to high levels of AMR [8]. Multidrug resistance (MDR) in NTS is particularly concerning, as resistance to three or more antimicrobial classes can result in reduced options for treatment of invasive disease and affect future therapeutic outcomes [9].

Culturing of faecal samples for pathogen identification and characterisation is common in clinical settings [10]. More recently, genomic approaches using sequence data from isolates have facilitated improved pathogen typing and prediction of AMR, reducing the dependency on phenotyping [11]. However, culturing and sequencing can be time-consuming, expensive and not always feasible [5]. Metagenomic sequencing, which involves the culture-free extraction and sequencing of total microbial DNA from samples, permits an examination of the whole microbial community within the sample and can give useful indications for potential causative agents of diarrhoea. *Salmonella* infections have been shown to be associated with a reduction in the gut microbial diversity and an increase in the abundance of Enterobacteriaceae, which in itself may generate a diagnostic signal, but further studies have been called for to better understand the interaction of *Salmonella* with the gastrointestinal microbiota [12]. Sequencing to sufficient depth can also facilitate the identification of specific causative agents of diarrhoea and the bacterial community resistome to guide treatment.

Here, we aimed to investigate the effectiveness of metagenomics for the identification of NTS, associated AMR genes and microbial community differences in faecal metagenomes collected from children with culture-confirmed salmonellosis in Vietnam. A variety of different informatics approaches were compared to attempt to identify the culture-confirmed *Salmonella* serovar from each metagenome: taxonomic profiling of metagenome reads, alignment of reads to a *Salmonella* reference database followed by local assembly and realignment, *Salmonella in silico* typing resource (SISTR) classification of local assemblies, and *de novo* assembly and metagenome-assembled genome (MAG) identification and classification. AMR genes were also identified from the reads and MAGs. The metagenomes from infected cases were compared to those from healthy controls with respect to the relative abundances of NTS, microbial composition and diversity and the resistome. We conclude that whilst *S. enterica* relative abundance was significantly higher in cases compared to controls, there is an overlap that makes determining a diagnostic cutoff difficult. Detection of serovars and *Salmonella*-specific AMR genes in the metagenomes is challenging, requiring higher sequencing depth to allow for more accurate diagnostics.

## Methods

### Sample collection and ethical approval

This study investigated 28 stool samples from paediatric culture-confirmed cases of salmonellosis. Samples were collected at Children’s Hospital No. 1 in Ho Chi Minh City, Vietnam, from children under 5 years of age presenting with symptoms of enterocolitis. Sample collection took place either on the day of admission (*n*=15; before any antibiotic treatment) or up to 3 days after admission (*n*=13) and thus possibly after antibiotic treatment was initiated. Ethical approval for this study was provided by the institutional review board of the hospital and the University of Oxford Tropical Research Ethics Committee (OxTREC no. 1045–13). The metadata associated with the samples can be accessed in Table S1 (available in the online Supplementary Material).

### *Salmonella* isolation

All faecal samples were cultured on MacConkey agar (MC agar; Oxoid) and xylose-lysine-deoxycholate agar (Oxoid), as well as in selenite broth (Oxoid) at 37 °C for 18–24 h. *Salmonella* isolates (*n*=28; one per sample) were identified based on their distinctive appearance on xylose-lysine-deoxycholate and MC agar, and their identification was confirmed using MALDI-TOF MS (Bruker) and API20E (bioMérieux), following the manufacturer’s instructions.

### DNA extraction, whole-genome sequencing and metagenome sequencing

The genomic DNA was extracted from all 28 isolates using the Wizard Genomic DNA Purification Kit (Promega), and whole-genome sequencing was performed at the Wellcome Sanger Institute. Extracted DNA of *Salmonella* isolates was sequenced on the Illumina HiSeq X Ten platform according to the manufacturer’s protocols to generate 150 bp paired-end reads.

Metagenome DNA from faecal samples was extracted using the FastDNA Spin Kit for Soil (MP Biomedicals), according to the manufacturer’s instructions. Metagenome sequencing was also performed at the Wellcome Sanger Institute, using the Illumina HiSeq 2500, generating 125 bp paired-end reads.

The sequence data are available in the National Center for Biotechnology Information (NCBI) Sequence Read Archive and European Nucleotide Archive (ENA) under project accession PRJEB21259.

### Genome analysis

Analyses were performed for all 28 isolate genomes using Galaxy [13], a QIB cloud server (based on Cloud Infrastructure for Microbial Bioinformatics [14]) and the Norwich Research Park High Performance Computing cluster.

The Illumina paired reads were trimmed using fastp v0.23.2 [15]. Reads were assembled with Shovill v.1.1.0+galaxy0 (https://github.com/tseemann/shovill) using the SPAdes assembler [16] with an estimated genome size of 5 Mbp and minimum coverage to call part of a contig set to 0 (AUTO), and assembly quality was assessed with QUAST v5.0.2 [17] and CheckM v1.0.11 [18]. BWA-MEM Galaxy v0.7.17.1 [19,20] and CoverM v0.3.2 (https://github.com/wwood/CoverM) were used to estimate contig coverage. Assemblies that displayed >30× coverage, <500 contigs over 500 bp and <50 duplicate marker genes were accepted.

Assemblies were annotated with Prokka v1.14.5 [21], and Roary v3.13.0 [22] (95% identity threshold for blastp and 99% threshold for the percentage of total isolates a gene must be in to be considered a core gene) was used for core gene alignment. This was used to construct a maximum likelihood tree in IQ-TREE v1.6.11 [23] using a general time-reversible model on the web server (https://www.hiv.lanl.gov/content/sequence/IQTREE/iqtree.html) with ultrafast bootstrap approximation [24] and sH-like approximate likelihood ratio test (1,000 replicates) [25].

The species, subspecies, serovar and sequence types (STs) were determined with SISTR v1.0.2 [26] and MLST v2.16.1 (https://github.com/tseemann/mlst), respectively. AMR determinants were identified using KMA v1.4.3 [27] and the ResFinder database [28] with 90% query identity and template coverage thresholds. The *aac(6′)-Iaa* gene was considered to confer an aminoglycoside-resistant genotype, although this is a cryptic gene in *Salmonella* and may not typically confer phenotypic resistance [29]. StarAMR v0.4.0 was used to identify fluoroquinolone resistance mutations with the PointFinder database [30], using 90% blast identity and overlap thresholds.

### Metagenome analysis

#### Dataset description and read processing

The Illumina paired reads of the ‘case’ faecal metagenomes (*n*=28) were each associated with one isolate genome from the ‘Genome analysis’ section (Table S2). The metagenome read files for individual runs were concatenated (merged) to obtain one pair of files per sample prior to analysis. ‘Control’ faecal metagenomes (*n*=21) from healthy children were obtained from a study by Pereira-Dias *et al*. [31] and downloaded from the ENA project PRJEB22032 (Table S3). These control samples were obtained from the Oxford University Clinical Research Unit at the Hospital for Tropical Diseases, also in Ho Chi Minh City, from children aged up to 5 years.

Reads were trimmed using fastp v0.23.4 and host reads removed with Hostile v0.1.0 [32] using the T2T-CHM13 v2.0+IPD-IMGT/HLA v3.51 human reference genome. Further analysis was performed on the trimmed, host-depleted reads.

#### Classification of reads for family-, genus- and species-level metagenome profiling, identification of *S. enterica* and *S. enterica* serovars

The reads were classified with Kraken2 v2.1.1+galaxy0 [33] (k2_nt_20230502 with 0.1 confidence level) and the abundance of species, genera and families estimated with Bracken v2.8 [34] (100mer k-mer distribution, read threshold to include as part of Bracken report set to 10). The 20 most abundant species and genera and 10 most abundant families in the metagenomes were determined using the Bracken outputs, by dividing the new_est_reads in Bracken reports by the number of overall metagenome reads obtained from Kraken reports (sum of unclassified and root), and multiplying by 100 to obtain percentages. *S. enterica* relative abundances were calculated in the same way.

Reads were also classified with Centrifuge v1.0.3 (nt_2018_3_3 database, with -k set to 1) [35]. The Centrifuge outputs were converted to Kraken-style reports using Centrifuge k-report to quantify *S. enterica* reads. The percentage of the overall faecal microbiome represented by *S. enterica* was calculated as described above.

MetaPhlAn4 v4.0.3 (mpa_vJan21_CHOCOPhlAnSGB_202103) [36] was also used for classification, as an alternative marker gene-based method. MetaPhlAn outputs were combined into one report using the merge_metaphlan_results.py script, and the *S. enterica* relative abundances were reported directly.

Centrifuge results were also used to identify reads representing the isolated *S. enterica* serovar for the case metagenomes, and the most abundant *S. enterica* serovar for the case and control metagenomes. For the detection of monophasic *Salmonella* Paratyphi B var. Java, the serovar was considered to be present regardless of monophasic or biphasic *S*. Java identification. However, for the monophasic variant of *S*. Typhimurium (I 4,[5],12:i:-), the metagenomes were specifically screened for *S*. 4,[5],12:i:-, as this variant was present in the databases used. The relative abundance of *S. enterica* serovars was calculated in the same way as species from the Centrifuge k-reports.

#### Alpha and beta diversity analysis

The alpha and beta diversities of the case and control metagenomes were assessed using the genus-level Bracken reports as input to make an operational taxonomic unit (OTU) table using R v4.2.3 [37]. Bracken by default only retains genera with at least 10 reads; thus, only those genera were included. NA values representing either absent taxa or taxa with fewer than 10 reads were changed to 0 for the analysis.

For alpha diversity, the metagenomes were rarefied to the smallest library size (28,644) with ‘rrarefy’ from vegan v2.6–4 [38]. Observed richness was calculated manually by counting the number of genera with non-zero values in each metagenome. Shannon and Simpson diversity indices were calculated using vegan.

Beta diversity analysis was also performed using vegan. The OTU table was transformed such that each genus abundance was divided by the sum for the sample to obtain relative abundance. This was used to calculate Bray–Curtis distance, which was used for non-metric multidimensional scaling (NMDS) in two dimensions (trymax=100).

#### Alignment of reads to the *Salmonella* database

NCBI (https://www.ncbi.nlm.nih.gov/) was searched for *Salmonella* reference genomes representing different serovars; 117 were identified and used to form a database after removing plasmid contigs. BBsplit v38.75 (https://github.com/BioInfoTools/BBMap/blob/master/sh/bbsplit.sh) was used to align the reads of each metagenome against this database and extract the *Salmonella* reads, which were assembled using SPAdes v3.14.1. The assembled contigs were aligned back to the database with Nucmer v3.1 [39] using 95% identity and coverage thresholds. The proportion of each chromosome in the database to which the contigs aligned was calculated. The serovar displaying the highest coverage was defined as the most likely serovar present. The assemblies were also analysed with SISTR core genome multilocus sequence typing (cgMLST) to determine the most likely serovars present.

#### Assembly of contigs and MAGs

Reads were assembled into contigs using MEGAHIT v1.1.2 [40] and coverage estimated with Bowtie2 v2.3.4.1 [41] and CoverM. MAGs were assembled using Metabat2 v2.14 [42] and Maxbin2 v2.2.4 [43]. MAG quality was assessed with QUAST [17] and CheckM [18]. BAT v5.0.3 [44] was used to identify *Salmonella* MAGs; the serovar of these was then classified with Kmerfinder v3.0.2+galaxy0 (https://cge.food.dtu.dk/services/KmerFinder/) using the bacteria database (compiled 17 October 2021) [27,45, 46]. *Salmonella* MAGs displaying less than 10% completeness were discarded.

#### Detection of AMR genes in metagenome reads and MAGs

KMA was used for AMR gene identification, using ≥90% query identity and ≥60% template coverage thresholds. The isolate genome and metagenome KMA results were compared to determine whether or not *S. enterica* AMR genes were identified in the associated metagenomes. AMR gene variant names were collapsed at the root, such that multiple variants were considered as one unique gene and a gene was considered to be present in the associated metagenome regardless of the specific variant. *gyrA* mutations were excluded when calculating the percentage of isolate genome AMR determinants identified in the associated metagenome, as these could not be identified in the metagenomes. The relative abundance of each AMR gene in the metagenome was calculated by dividing the number of bases associated with the gene (calculated by multiplying the KMA template length and the depth) by the total number of bases associated with AMR genes in that sample and then displayed as a percentage. The metagenomes were then screened for the AMR genes identified in the *Salmonella* isolate genomes. Relative abundances of these genes were summed up to determine the relative abundance of *S. enterica* AMR genes in each metagenome. For genes with multiple variants, the proportions of all variants were added up. The classes of antimicrobials to which the genes confer resistance were reported according to the Comprehensive Antibiotic Resistance Database (CARD) [47] (Table S4) to identify AMR gene classes, except for beta-lactamase genes, for which the antimicrobial class was simplified to ‘beta-lactams’. The drug class for *mcr* genes was changed to polymyxins instead of the peptide classification given by CARD, as this is a more distinct representation of the resistance class [48]. For genes that were not present in the CARD database, the AMR gene classes were obtained from published literature. The gene classes were summarised as ‘multiple’ for genes conferring resistance to more than one antimicrobial class. The class summary was used for plotting or analysis unless stated otherwise.

The accepted Metabat2 and Maxbin2 MAGs were analysed with ABRicate v0.9.7 (https://github.com/tseemann/abricate) using the ResFinder database and 90% identity and 60% coverage thresholds. MAG contigs containing AMR genes were mapped against publicly available sequences with blastn v2.15.0+ (megablast) [49] using the nucleotide collection (nr/nt, updated 12 January 2024) [50] on the online server (https://blast.ncbi.nlm.nih.gov/Blast.cgi) with default parameters. Contigs containing *aac(6′)-Iaa* genes were not mapped as these genes are considered cryptic (not conferring phenotypic resistance) in *Salmonella* [29]. From the first 100 chromosome and plasmid hits, those with ≥90% identity were reported.

#### Statistical analysis

Statistical analysis was performed in R. The relative abundance of *S. enterica* and Enterobacteriaceae in the case and control metagenomes, the percentage of metagenome reads aligned to the custom *Salmonella* database and the percentage of reference chromosome covered following assembly and realignment (top result per metagenome), as well as the number of total AMR variants, unique AMR genes and number of antimicrobial classes to which the genes confer resistance, were compared using Mann–Whitney *U* tests. The alpha diversity indices of case and control metagenomes were also compared using Mann–Whitney *U* tests. Fisher’s exact tests were used to compare the proportions of metagenomes containing genes conferring resistance to AMR classes commonly used in *Salmonella* treatment; this included genes conferring resistance to multiple antimicrobial classes where relevant.

Kendall’s correlation tests were performed to investigate whether or not the ability to detect *S. enterica* or the isolated serovar and its AMR determinants in the associated metagenome was associated with sequencing depth.

It was assumed that higher relative abundances of *S. enterica* reads in the metagenome would increase the likelihood of detection of the isolated *S. enterica* AMR determinants. The relative abundance of *S. enterica* in the metagenomes and the percentage of *S. enterica* AMR genes identified in the case metagenomes were compared using Kendall’s correlation to test this formally.

For beta diversity analysis, centroids were compared using PERMANOVA (adonis2), and heterogeneity of dispersions was assessed with betadisper and permutest from vegan.

A significance level threshold of 0.05 was used for all statistical tests.

## Results

### *Salmonella enterica* isolated from diarrhoeic stool samples

The 28 genomes representing isolates collected from salmonellosis patients were classified as *S. enterica* subsp. *enterica*. A total of 11 different serovars and 13 associated STs were identified (Table 1).

**Table 1.** The serovars and STs identified amongst the 28 cultured *S. enterica* isolate genomes

Serogroup	Serovar	ST	No. of isolate genomes
B	Typhimurium	34	2
		19	2
		36	1
	Monophasic Typhimurium (I 4,[5],12:i:-)	34	11
	Stanley	29	1
		2615	1
	Paratyphi B var. Java monophasic	423	1
	Saintpaul	50	2
C2–C3	Newport	4157	1
	Albany	292	1
D1	Enteritidis	11	2
E1	Weltevreden	365	1
E4	Meleagridis	463	1
I	Hvittingfoss	446	1

**Fig. 1.**
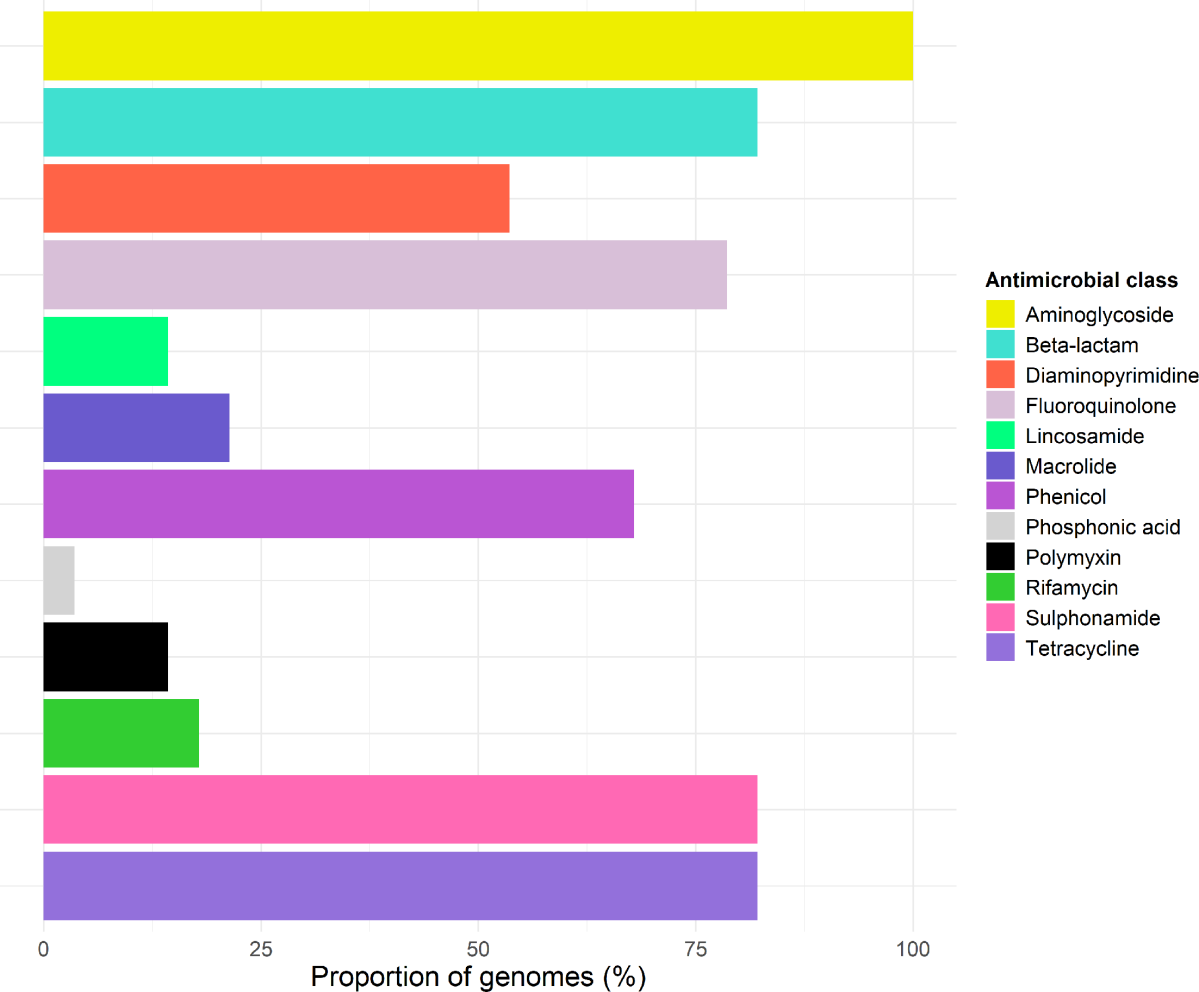
Distribution of the percentage of 28 case-associated *S. enterica* isolate genomes containing genes conferring resistance to specific antimicrobial classes.

Thirty-five unique AMR determinants, including genes and resistance-associated mutations, were identified amongst the 28 *Salmonella* isolate genomes with KMA and starAMR (Table S5). The AMR determinants identified conferred resistance to 12 different antimicrobial classes (Fig. 1). A high proportion of isolate genomes (82.1%) exhibited a MDR genotype (predicted resistance to three or more antimicrobial classes). The number of unique AMR determinants per genome ranged between 1-18 (median=9).

### Microbial communities within case and control metagenomes

The total number of reads obtained for the metagenomes after trimming and host read depletion ranged between 30,576 and 94,890,832 for the case metagenomes and 10,821,636 and 15,177,561 for the control metagenomes.

**Fig. 2.**
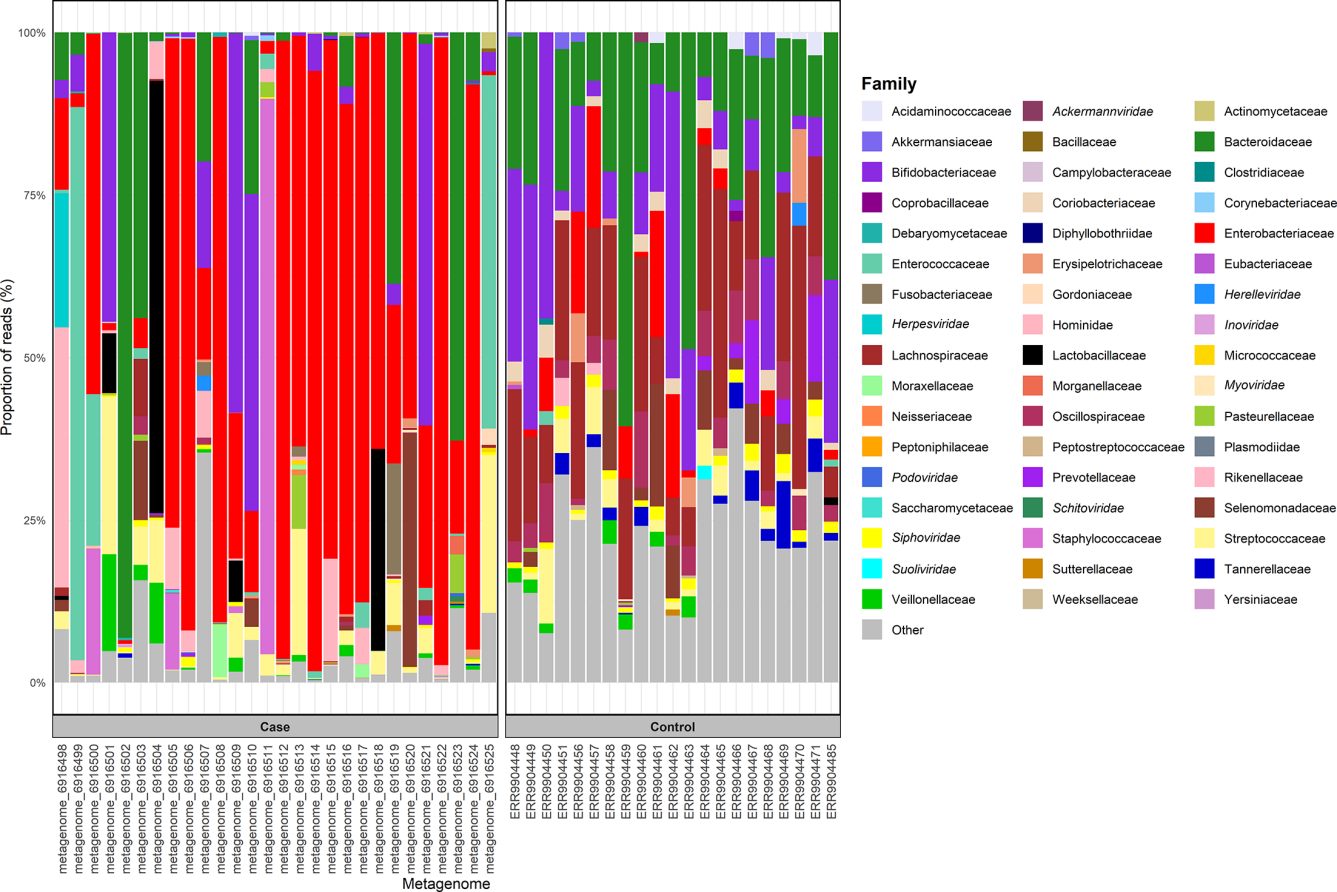
The ten most abundant families in each case and control metagenome based on Bracken results, with remaining classifications grouped into the ‘other’ category in each sample.

The ten most abundant families in the faecal samples were compared and represented 64.6–99.6% (median 97.7%) and 57.9–92.5% (median 78.2%) of the overall case and control metagenomes, respectively. The most dominant microbial family amongst the case metagenomes was Enterobacteriaceae, representing the most abundant family in 14 samples (Table S6). The relative abundance of Enterobacteriaceae was significantly higher (*P*=7.66×10^−5^) in the case metagenomes (0.219–96.6%, median=40.2%) compared to controls (0.0246–19.4%, median=1.47%). Within this, *Escherichia* was the most prevalent genus in case metagenomes (Fig. S1), along with *Klebsiella,* and *Escherichia coli* was the most dominant species (Figs S2 and S3, Table S7). A smaller number of case metagenomes were predominated by Bacteroidaceae (*n*=5), Bifidobacteriaceae (*n*=4), and to a lesser extent other microbial families, whereas control metagenomes displayed more diverse and uniform microbial profiles, with Lachnospiraceae being the most abundant family in eight samples, followed by Bacteroidaceae, Bifidobacteriaceae and Enterobacteriaceae. At the genus level, the control metagenomes were predominated by *Bifidobacterium* and *Bacteroides*. Taken together, this suggests less diversity in the case metagenome group compared to controls, with the top ten most abundant families representing a larger proportion of the overall population (Fig. 2).

**Fig. 3.**
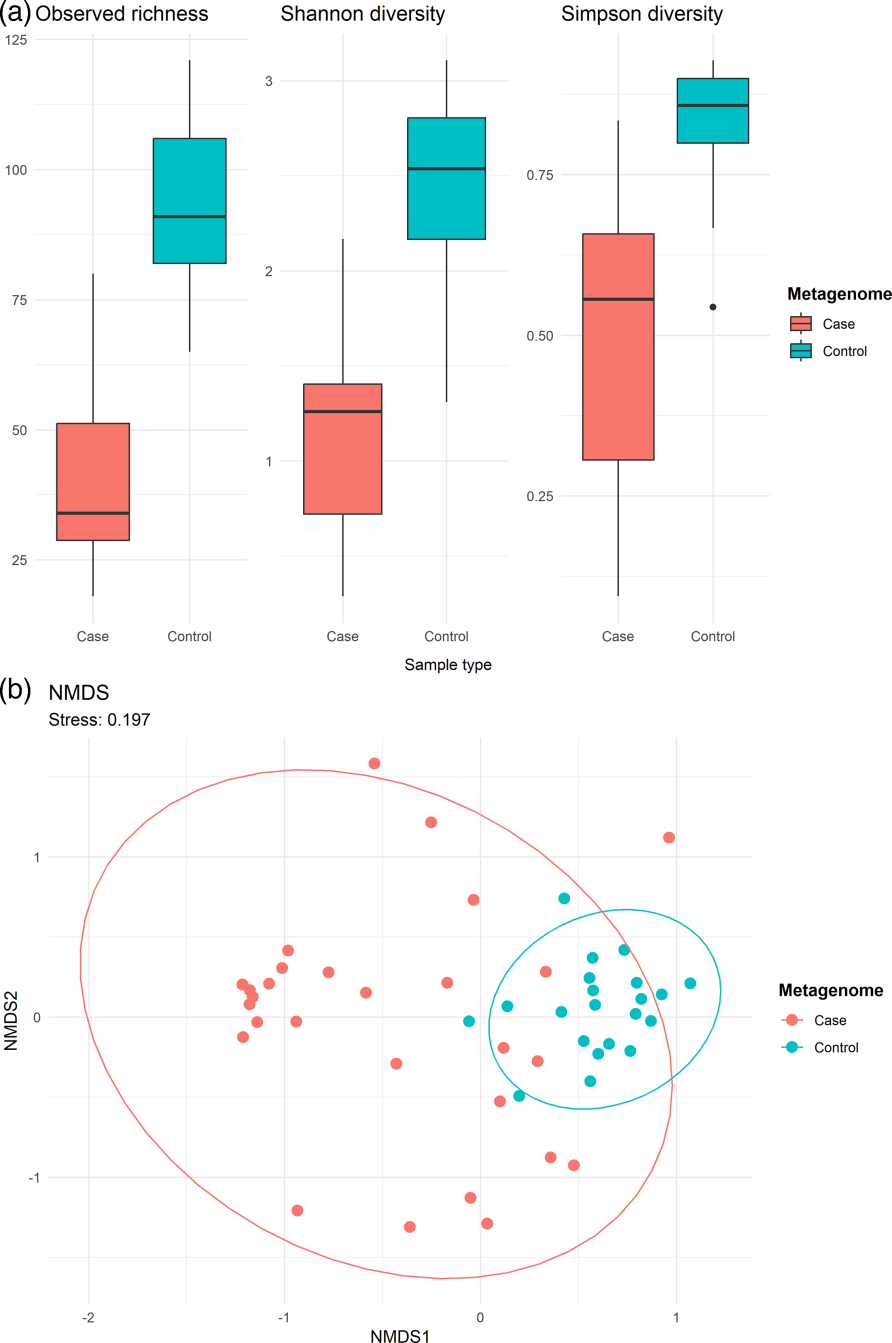
Alpha and beta diversity analyses of the case and control faecal metagenomes. Observed richness, Shannon and Simpson diversity indices calculated from Bracken results at the genus level for the case and control metagenomes, after rarefying to the smallest sample size (**a**); NMDS plot displaying dissimilarity between case and control metagenomes based on Bray–Curtis distance calculated from genus relative abundances from Bracken (**b**).

To explore microbiome differences between sample groups further, alpha and beta diversities of case and control metagenomes were investigated. There was a significant difference in the observed richness (*P*=2.31×10^8^), Shannon (*P*=2.31×10^10^) and Simpson (*P*=4.09×10^9^) diversity indices between the sample types (Fig. 3a), with the control group displaying higher alpha diversity compared to case metagenomes. There was also a significant difference in the beta diversity between case and control metagenomes (adonis2 *P*=0.001). Analysis of the heterogeneity of dispersions revealed that the variances of the sample groups also differed significantly (*P*=0.001), which was reflected in the NMDS plot (Fig. 3b). The control samples clustered closely together, whereas the case metagenomes were more dispersed. Overall, the case metagenomes displayed less diversity and more heterogeneity with a predominance of select microbial families compared to controls.

### Detection of *S. enterica* and isolated serovars in the associated faecal metagenomes

To assess the utility of metagenomics as a tool to identify diarrhoea-causative *Salmonella* directly from culture-confirmed stool samples, the percentage of reads represented by *S. enterica* in the case metagenomes was determined and compared to the percentage of reads represented by *S. enterica* in the healthy control stool metagenomes.

**Fig. 4.**
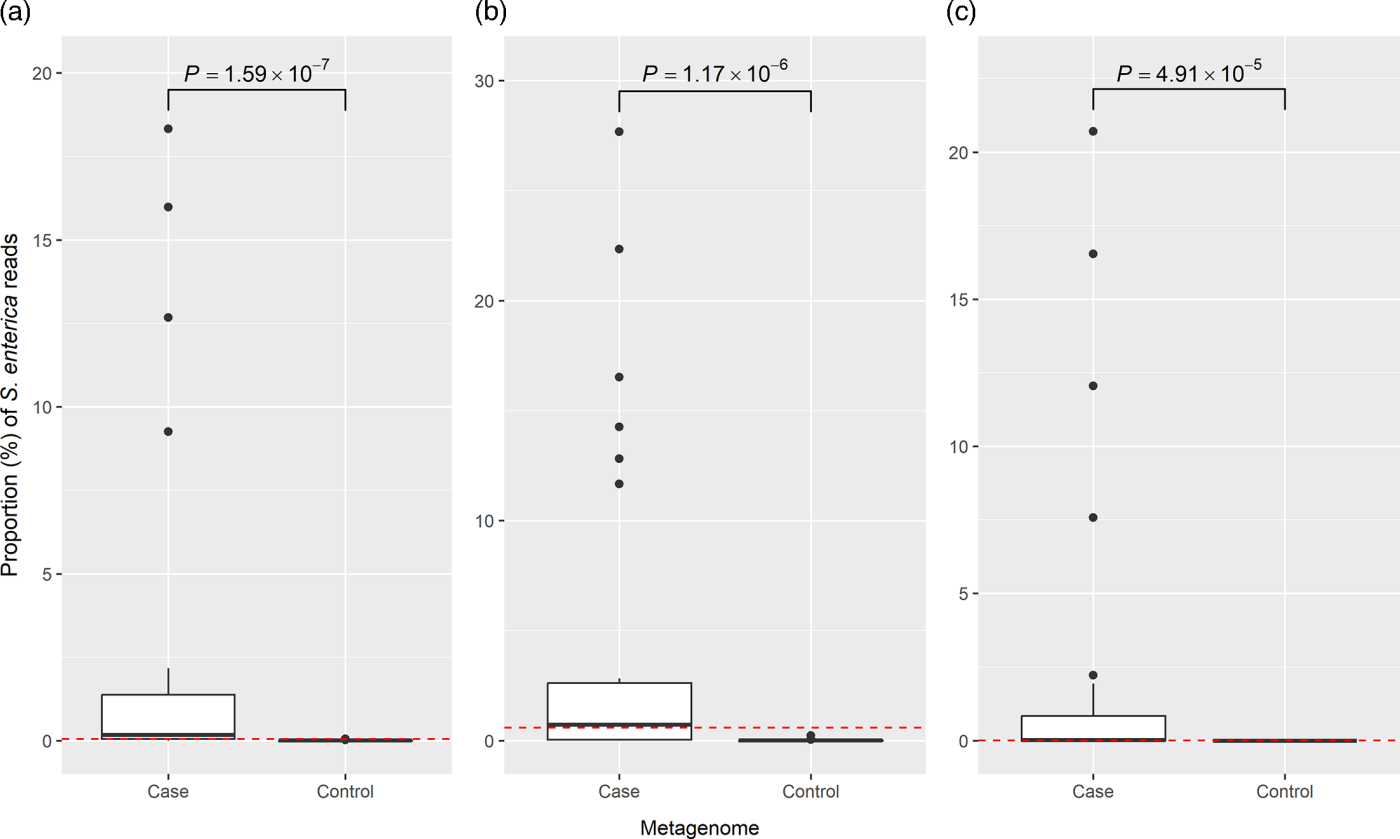
Proportion of case and control metagenome reads represented by *S. enterica* with Centrifuge (**a**), Bracken (**b**) and MetaPhlAn (**c**), with the Mann-Whitney *U P*-value displayed above the boxplots, and the lowest proportion associated with only case metagenomes highlighted by the red, dashed threshold line.

**Fig. 5.**
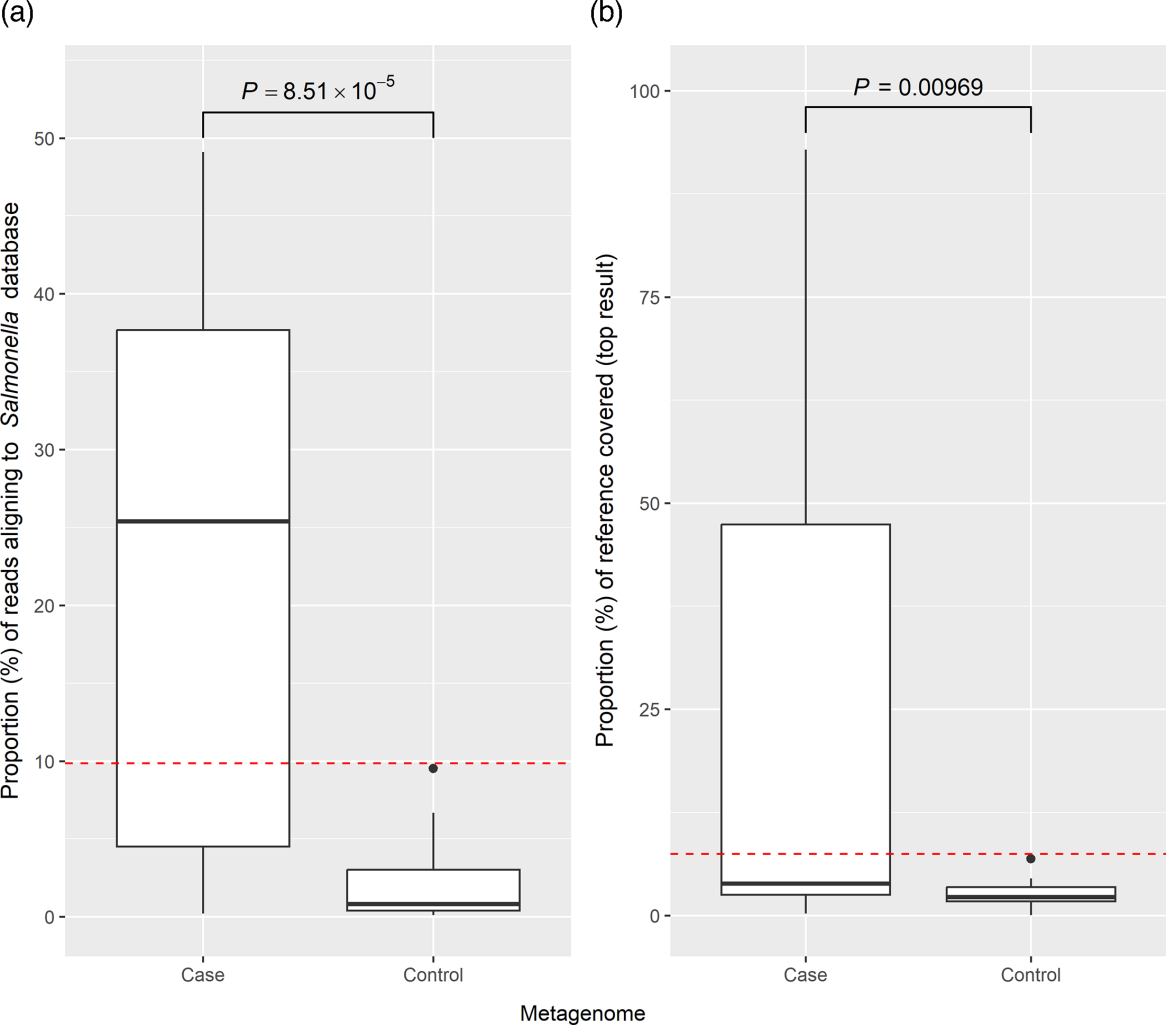
The proportion of metagenome reads aligning to the custom *Salmonella* serovar database (**a**) and the highest proportion of reference chromosome covered after assembly of the aligned reads and realignment to the custom *Salmonella* serovar database (**b**), with the Mann-Whitney *U P*-value displayed above the boxplots, and the lowest proportion associated with only case metagenomes highlighted by the red, dashed threshold line.

*S. enterica* was identified in all 28 faecal case metagenomes with Centrifuge and Bracken. However, the percentage of the metagenome reads represented by *S. enterica* was variable (Fig. 4, Table S8). There was no significant association (*P*=0.150–0.298) between the percentage of *S. enterica* and the total number of metagenome reads. *S. enterica* was identified in all 21 of the control metagenomes with Centrifuge and 17 (81.0%) control metagenomes with Bracken (Table S9). The percentage of *S. enterica* reads in the case and control metagenomes varied significantly for both the Centrifuge (*P*=1.59×10^−7^) and Bracken (*P*=1.17×10^−6^) methods (Fig. 5). However, the distribution of *S. enterica* relative abundance in the case and control metagenomes was not entirely distinct, with some overlap at lower case percentages. Relative abundances of at least 0.0581% and 0.598% with Centrifuge and Bracken, respectively, were associated exclusively with case metagenomes, although there was a difference in the relative abundances for individual samples determined following analysis with the two tools (Tables S8 and S9). Sixteen case metagenomes were at or above the Bracken threshold, and 21 were at or above the Centrifuge threshold. All of the metagenomes above the Bracken threshold were also above the Centrifuge threshold. As an alternative approach, we also classified reads with MetaPhlAn4 to see whether or not this improved the discrimination between case and control metagenomes. With this approach, *S. enterica* was not detected in any of the control metagenomes (Table S9). However, the species was also not detected in 12 of 28 case metagenomes (Table S8). The lowest relative abundance of *S. enterica* detected with MetaPhlAn4 was 0.00577%, indicating that the potential diagnostic threshold varies by classification method.

The specific serovar of the isolated *S. enterica* was identifiable in 22 (78.6%) of the associated case metagenomes with Centrifuge (0.00000208–1.82 % of total reads, median=0.0000745%). There was no significant correlation between the percentage of the isolate genome serovar in the metagenome and the total number of metagenome reads (*P*=0.953). Despite being identifiable in a large proportion of metagenomes, the isolate genome serovar was the most abundant in only six (21.4%) case metagenomes based on Centrifuge results. *S. enterica* serovar sequences were also identified in all control metagenomes.

The metagenome reads were also mapped to a custom *Salmonella* database consisting of available reference genomes from NCBI, to facilitate serovar detection. The percentage of metagenome reads aligning to this database ranged between 0.200 and 49.1% for cases and 0.107 and 9.53% for controls (Fig. 5); these percentages were significantly different (*P*=8.51×10^−5^). Alignment percentages of at least 9.86% were associated exclusively with case metagenomes; this is considerably higher than the relative abundance thresholds for species relative abundance with taxonomic profiling. These reads were assembled and then aligned back to the genomes in the reference database. The serovar reference displaying the highest percentage of chromosome covered through alignment to a *Salmonella* reference database, assembly and realignment ranged between 0.243% and 92.8% (median=3.89%) in cases, though high initial read alignment percentage did not always result in a high percentage of reference chromosome covered (Fig. 5, Table S8). Nonetheless, the distribution of the highest percentage of chromosome covered was significantly different between cohorts (*P*=0.00969), with alignment proportions of at least 7.47% associated exclusively with case metagenomes. The isolated serovar was predicted as the most likely serovar present in 10 of the 28 case metagenomes with the Nucmer alignment method. SISTR analysis on the assemblies resulted in the correct prediction of the associated isolate genome serovar in eight case metagenomes (Table S8); however, it is important to note that the majority of assignments had low confidence as denoted by a QC warning or fail due to missing cgMLST loci, which was expected as this tool was not designed for use with fragmented assemblies from metagenomes. A SISTR cgMLST serovar prediction was also given for all controls.

**Fig. 6.**
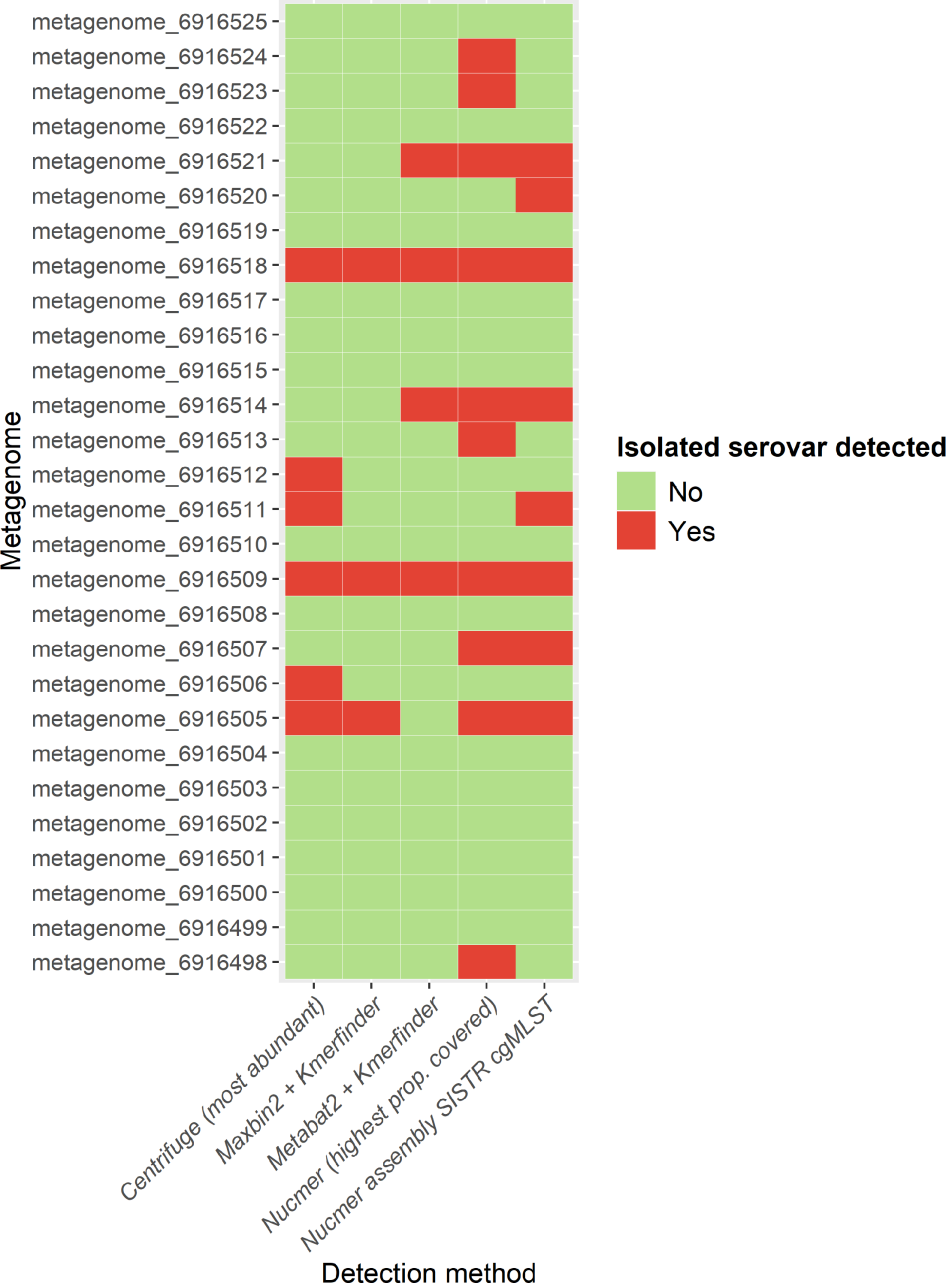
Summary of the detection (yes/no) of the isolated *S. enterica* serovar in the case metagenomes by each detection method used; for Centrifuge, the serovar was marked as detected if it was the most abundant serovar in the sample and for Nucmer if it displayed the highest proportion of the reference chromosome covered.

*Salmonella* MAGs with >10% completeness were assembled in six case metagenomes only. Metabat2 assembled 1–3 *S*. *enterica* MAGs in five samples (completeness, 12.5–75.9 %; contamination, 0.00–1.75 %), whereas Maxbin2 identified one *S. enterica* MAG in six samples (completeness, 14.7–99.2%; contamination, 0.00–3.03%). Accepted *Salmonella* MAGs were identified in samples with high *S. enterica* relative abundance or high sequencing depth (Table S8). Kmerfinder classifications of the MAGs revealed the associated isolate genome serovar in four and three metagenomes for Metabat2 and Maxbin2, respectively. Overall, alignment and assembly methods identified the serovar of the associated isolate genome in 12 samples (Fig. 6).

This indicates that the efficacy of detection of the isolated serovar also varies by method, though overall the identification of isolated serovars was less common than the pathogen species. *S. enterica* serovar Meleagridis was not identified in the associated case metagenome through any method. Identification was not possible with the serovar alignment method as a reference genome was not available in the database. Another metagenome (Metagenome_6916498) did not produce any MAGs.

### The resistome of case and control metagenomes and detection of AMR genes identified in the *S. enterica* isolate genomes through metagenomics

**Fig. 7.**
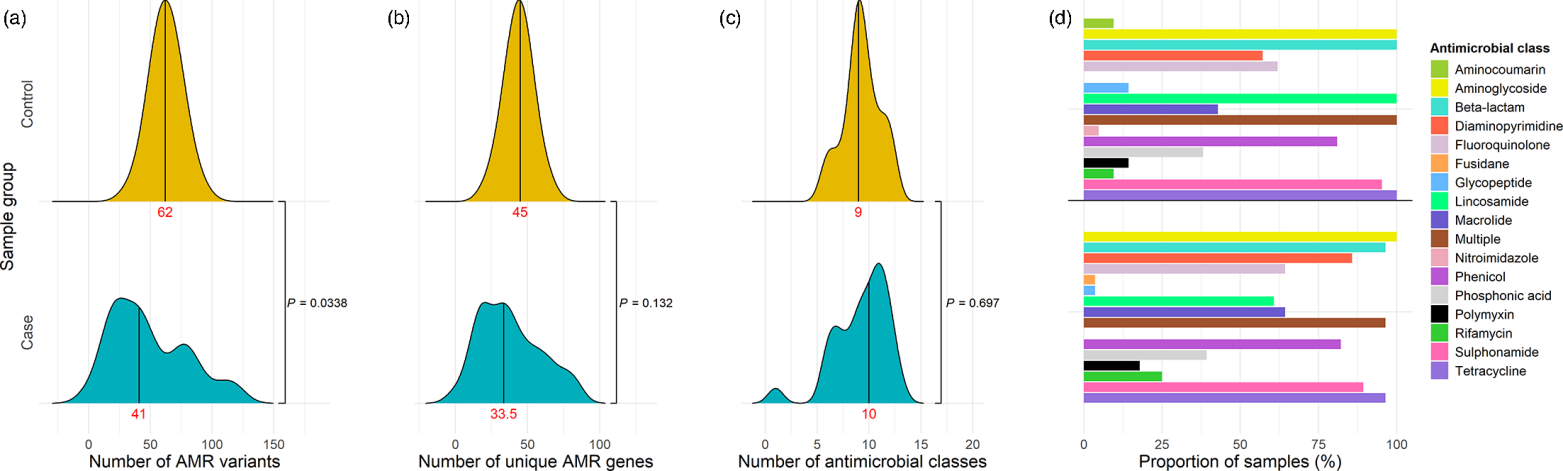
Comparison of the distribution of the number of individual AMR genes (**a**), unique genes (**b**) and antimicrobial classes represented by the AMR genes (**c**) identified in the case and control metagenomes with the median indicated in red, as well as the percentage of metagenomes containing genes conferring resistance to specific antimicrobial classes (**d**).

The resistome of the case and control metagenomes was explored. There was a significant difference in the number of total AMR variants between cases and control metagenomes (*P*=0.0338), though no significant difference between the number of unique AMR genes (*P*=0.132) nor the number of antimicrobial classes to which these genes were predicted to confer resistance (*P*=0.697, Fig. 7). Most of the resistance classes were observed in both metagenome groups, except fusidane resistance genes were only observed in the case group, whereas aminocoumarin and nitroimidazole resistance genes were only observed in the control group. The percentage of metagenomes with genes conferring resistance to diaminopyrimidines, glycopeptides, lincosamides, macrolides and rifamycins varied considerably between groups. However, the level of resistance to classes including macrolides and lincosamides may be partly represented by genes conferring resistance to multiple antimicrobial classes, which were observed in 96.4% of case and 100% control metagenomes. When comparing the proportion of samples with resistance genotypes for antimicrobial classes used in salmonellosis treatment, there was no significant difference between the proportion of case and control metagenomes with genes conferring resistance to beta-lactams (Fisher’s exact test, *P*=1), macrolides (*P*=0.5) or fluoroquinolones (*P*=1). Overall, this indicates similar resistomes between cohorts.

**Fig. 8.**
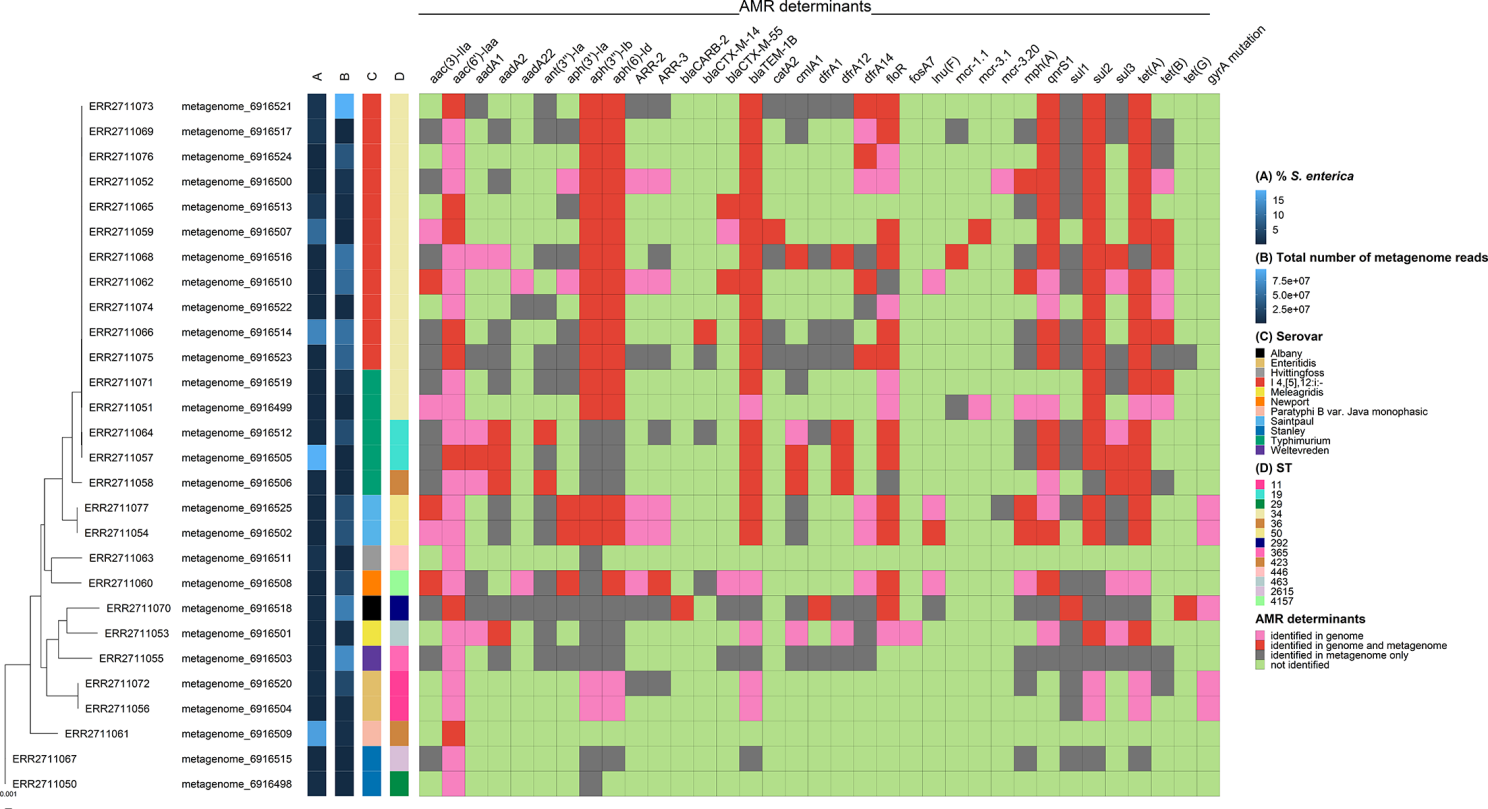
Maximum likelihood tree based on core gene alignment of the 28 *S. enterica* isolate genomes, with the associated metagenomes labelled, as well as the serovars, STs and the AMR determinants of the *S. enterica* isolate genomes identified with KMA indicated; the AMR determinant matrix also indicates whether or not the gene was identified in the associated faecal metagenome. (A) The percentage of *S. enterica* determined based on Centrifuge results, (B) overall number of reads in the metagenome, (C) serovar of the *S. enterica* isolate genome, (D) ST of the *S. enterica* isolate genome*.*

Characterisation of *S. enterica* AMR genotypes directly in stool metagenomes may be beneficial for guiding treatment in severe cases and invasive disease. Therefore, the metagenomes from the cases were screened for AMR genes identified in the associated *S. enterica* isolate genomes. The percentage of AMR genes in the isolate genomes identified in the associated metagenomes ranged between 0.00 and 100% (Fig. 8, Tables S5 and S10). All the unique AMR genes identified in the isolate genomes were also identified in seven (25.0%) of the associated metagenomes. There was a significant association between the percentage of unique isolate genome AMR genes identified in the associated metagenomes and the percentage of *S. enterica* reads in the metagenomes based on Centrifuge (*P*=0.00531) and Kraken2 and Bracken (*P*=0.000177) classification. However, the total number of metagenome reads did not significantly correlate with the percentage of AMR genes identified (*P*=0.113). Many genes commonly identified in the isolated *S. enterica* and the case metagenomes from this study were also identified in the control metagenomes (Figure S4, Table S11), except for *aac(6′)-Iaa*, *aadA22*, *bla*_CTX-M-55_, *lnu(F*), *mcr-3.1* and *mcr-3.20*. The *aac(6′)-Iaa* gene, a cryptic AMR gene found in all of the *S. enterica* isolate genomes investigated, was not detected in any of the control metagenomes. However, this gene was only identified in eight (28.6%) of the case metagenomes.

**Fig. 9.**
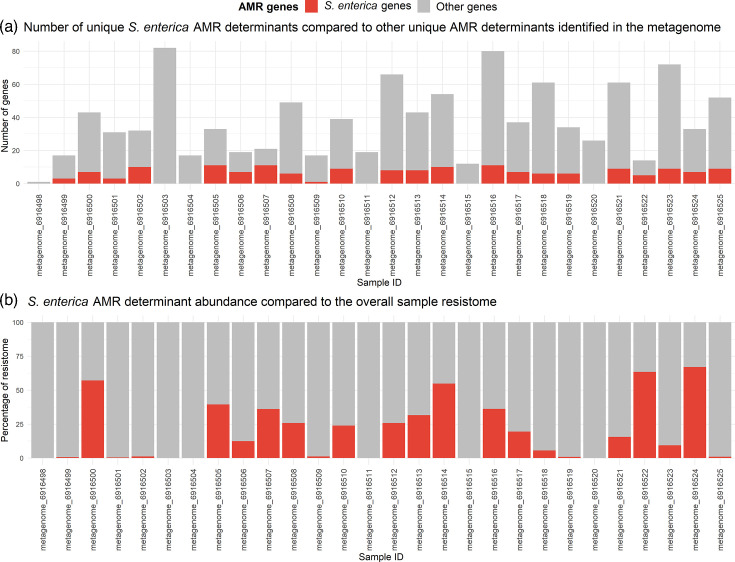
Comparison of the number of unique *S. enterica*-associated AMR genes and other genes present in the associated faecal metagenomes according to KMA (**a**) and the abundance of unique *S. enterica* AMR genes compared to the overall AMR gene abundance in the metagenomes (**b**).

The proportion of the sample resistome represented by *S. enterica* genes (AMR genes identified in the associated isolate genome) compared to other AMR genes present was investigated. The number of unique *Salmonella*-associated AMR genes identified in the metagenome ranged between 0 and 11 (Fig. 9a). The relative abundances of *Salmonella* AMR genes were calculated and compared to the overall AMR gene abundance in each sample (Fig. 9b). *Salmonella* AMR genes represented 0.00–67.1 % of the overall sample resistome, suggesting that although the number of genes commonly found in *Salmonella* is generally low compared to other AMR genes in the samples, the AMR genes associated with *Salmonella* can be highly abundant in the metagenome, which could potentially facilitate treatment guidance for salmonellosis cases. However, as most of the case metagenomes also contained high relative abundances of other Enterobacteriaceae, it is likely that the abundance of many of the *Salmonella*-associated genes was due to the genes also being present in related bacteria.

The MAGs obtained with Metabat2 and Maxbin2 were also screened for AMR genes, which resulted in the detection of 0–1 genes within the Metabat2 MAGs and 2–10 genes in the Maxbin2 MAGs (Table S12). The AMR gene identified in the Metabat2 MAGs (samples metagenome_6916509 and metagenome_6916514) was also identified in the *Salmonella* isolate genomes from those samples. However, the Maxbin2 MAGs from these samples contained *erm* genes that were not identified in the associated isolate genomes. This was also the case with other genes identified in the other Maxbin2 MAGs. The contigs of the Maxbin2 MAGs containing AMR genes were screened with blastn to investigate if sequences from other bacteria were incorporated into the MAGs. This revealed that the contigs containing *mecA* and *erm* genes were mostly associated with Gram-positive bacteria. Other genes (*dfrA12*, *tet(M)*, *qnrB4*, *ant(3″)-Ia*, *bla*_DHA-1_, *bla*_CTX-M-15_, *bla*_CTX-M-27_, *aph(3′)-Ia*, *lnu(F*), *mef(B*) and *bla*_CTX-M-55_) were often associated with plasmids or chromosomes of bacteria in the Enterobacterales order, including *Salmonella*. The AMR genes identified in the isolated *S. enterica* were always associated with Enterobacterales plasmids or chromosomes or other Gram-negative bacteria. This highlights that the MAGs from this study may not accurately reflect isolated *Salmonella* AMR genes.

### Identification of other diarrhoeagenic pathogens

In 12 case metagenomes, other potential diarrhoeagenic bacteria (*Shigella flexneri*, *Shigella boydii*, *Shigella dysenteriae*, *Shigella sonnei*, *Staphylococcus aureus*, *Clostridium perfringens* and *Clostridioides difficile*) were identified amongst the 20 most abundant species based on Bracken analysis. *Shigella flexneri* was identified in 10 case metagenomes with the relative abundance ranging between 0.0599 and 5.16 %, *Shigella boydii* in 8 (0.0198–0.694 %), *Shigella dysenteriae* in 5 (0.0251–0.485 %), *Shigella sonnei* in 4 (0.0295–0.132 %), *Staphylococcus aureus* in 4 (0.701–19.7 %), *Clostridium perfringens* in 1 (0.0720%) and *Clostridioides difficile* in 2 (0.193–0.265 %). *Clostridioides difficile* was also identified amongst the 20 most abundant species in 1 control (0.687%) metagenome. In 6 case metagenomes, at least one of the *Shigella* species identified amongst the 20 most abundant bacteria was more abundant than *S. enterica*, and in one case metagenome *Staphylococcus aureus* was more abundant than *S. enterica*. This highlights potential co-infection cases amongst the case metagenomes.

## Discussion

In this study, we aimed to determine if metagenomics can be used as a potential diagnostic tool to identify *S. enterica*, associated AMR genes for treatment guidance and microbial community changes associated with infection, using faecal metagenomes from culture-confirmed paediatric salmonellosis cases.

We were able to specifically identify *S. enterica* using metagenomics in all 28 case metagenomes with both Bracken and Centrifuge. Although *S. enterica* reads were also detectable in 81% and 100% of 21 control metagenomes obtained from individuals without diarrhoeal symptoms for at least 6 months prior to sampling with Bracken and Centrifuge, respectively [31], the relative abundance was significantly lower compared to cases. An overlap between cases and controls could indicate asymptomatic carriage in some controls, which has been previously reported in Vietnam [51]. The control samples were screened with the Luminex xTag Gastrointestinal Pathogen Panel Kit to determine the absence of pathogens [31], which is a screening kit for nucleic acids of gastrointestinal pathogens or their toxins. However, this assay has previously shown 83.3–92.3 % sensitivity in the detection of *Salmonella* spp. from clinical samples [52,54], thus suggesting the possibility of false negative results.

Low *S. enterica* relative abundances were also observed in a number of case metagenomes, though such observations in symptomatic cases are not unusual and could be related to different stages of infection, the progression of which can be affected by the innate gut microbiome [12,55]. Coinfections could also explain lower NTS abundance and have been previously observed in paediatric gastroenteritis cases in Southeast Asia [53]. Indeed, a number of other bacterial pathogens were identified amongst the most abundant taxa in the case samples. However, definitive metagenomic identification of certain pathogens, including *Shigella*, is challenging as *Shigella* is difficult to distinguish from *Escherichia* [56]; therefore, these results should be interpreted with caution. Similarly, the presence of bacterial toxins associated with gastroenteritis was not evaluated; thus, identification of organisms like *S. aureus* and *C. difficile* in the case metagenomes provides insufficient evidence of co-infection with these organisms. Taken together, these findings highlight that verification of a diagnostic threshold for *S. enterica* abundance using metagenomics may require further investigation in a larger study that accounts for these potential confounders.

A combination of methods was used to identify the serovar of the *S. enterica* isolated from the case metagenomes and pathogen-associated AMR genes for treatment guidance, which may be particularly important for paediatric cases that are at increased risk of progressive disease [57]. Although reads representing the isolated serovar could be identified in up to 78.6% of case metagenomes, the isolated serovar was the most abundant in only six (21.4%) case metagenomes according to Centrifuge, and *Salmonella* serovars were also detectable in all control metagenomes. The prediction capability of the alignment and local assembly, as well as SISTR serovar prediction, may be related to the *S. enterica* relative abundance in the case samples, although higher *S. enterica* relative abundance did not always result in the prediction of the isolated serovar. We observed significantly higher percentages of reads aligned to the custom *Salmonella* database in cases compared to controls, though after assembly of the aligned reads and realignment back to the reference database, the highest percentage of chromosome covered was not necessarily as high; this could be due to initial alignment of non-specific regions, which can be an issue in samples containing high abundances of other Enterobacteriaceae. The reliability of serovar characterisation with these methods is, therefore, limited in the absence of culture and genomic data. Use of methods that take into account coverage depth and breadth of alignment could help to resolve this in future studies [58].

The mapping approach used for detecting AMR genes showed similar indications. The percentage of the *Salmonella*-associated AMR genes identified was correlated with the *S. enterica* relative abundance and the unique genes identified in the *Salmonella* isolate genomes represented up to 67% of the sample resistome. However, many of the genes identified were likely carried by multiple organisms present in cases and also controls, as the resistomes were overall not significantly different between the cohorts. The identification of *S. enterica*-specific genes, such as *aac(6′)-Iaa*, was possible in only 28.6% of case metagenomes, indicating that mapping approaches may be insufficient to accurately characterise the resistome of individual pathogens within samples [59]. MAGs could provide this capability, but they can be contaminated with sequences from other organisms [60], which could help to explain why many of the AMR genes found in the MAGs were not identified in the *Salmonella* isolate genomes associated with the samples. This was particularly apparent for the Maxbin2 MAGs, the contigs of which often mapped to distantly related bacteria or plasmids of Gram-negative bacteria, including Enterobacterales, but not exclusively *Salmonella*. However, it is important to note that the threshold for *Salmonella* MAG completeness in this study was relatively low (10%) compared to the recommended threshold for medium- and high-quality MAGs [61], and a contamination cutoff was not applied, which could partly explain the lack of consistency between MAGs and isolate genomes. Higher sequencing depth and the use of long-read sequencing could improve MAG assembly and thus detection of NTS serovars and associated AMR genes in the metagenomes with increased confidence.

In the current study, the detection of *S. enterica* and *S. enterica* AMR genes in the associated metagenomes was largely reliant on the genomes of *S. enterica* from isolates recovered from the faecal samples. It is possible that the faecal samples contained more than one serovar or strain of *S. enterica* that was not detected with culture and genomics. This could explain why the Centrifuge results indicated that a different serovar was more abundant than the recovered serovar in multiple metagenomes. Detection of more than one *Salmonella* serotype from faecal samples has been previously documented [62,63]; similarly, multiple strains displaying differing gene repertoires within the same serovar can be recovered from one faecal sample [64]. However, as the practice of selecting multiple isolates per sample is generally rare, it is not possible to accurately determine the frequency of such occurrences.

The salmonellosis cases were associated with a marked disturbance in the faecal microbiome compared to controls, as indicated by significant differences in alpha and beta diversity. The case metagenomes were marked with significantly fewer genera than controls and the controls clustered closer together than case metagenomes, suggesting reduced diversity in the gut microbiome and a shift away from a more stable microbial community in the NTS cases. Reduced microbial alpha diversity has been previously observed in diarrhoea cases [65], and the microbiomes of infants with diarrhoea have been shown to display wider variation than healthy controls [66].

We specifically observed a significant increase in the relative abundance of Enterobacteriaceae in case metagenomes, mostly associated with *E. coli* and *Klebsiella* species. These species can replicate in the conditions created through inflammation initiated by *S. enterica* [67], though such changes have been reported across a range of different bacterial aetiologies of diarrhoea [68,69]. Increases in the abundance of other facultative anaerobes, such as *Enterococcus* [70], can also occur under these conditions, particularly in dysenteric diarrhoea, as observed in some samples in the current study. We hypothesise that the differences in the microbiome profiles between salmonellosis cases could be used to characterise different stages of infection, with increased relative abundances of facultative anaerobes indicative of later infection stages in which the conditions allow proliferation of specific taxa. Conversely, metagenomes with less observed microbiome disturbance could represent earlier stages of infection, as more normal microbiome profiles were observed in a minority of cases here and reported elsewhere [65], or, alternatively, later stages in which the microbiome begins to return to a more stable state [65]. This was also reflected in the beta diversity NMDS plot, which suggested some overlap between cases and controls. However, such inferences are complicated by the observation of increased levels of Enterobacteriaceae in some control samples from healthy individuals, though this again could indicate asymptomatic *S. enterica* carriage. This highlights the limitations associated with microbiome characterisation in infectious disease, but also reinforces the need for further evaluation of potential diagnostic thresholds and additional studies to understand the microbiome dynamics of symptomatic and asymptomatic individuals. Alternative methods based on symptom history may be currently more feasible for infection staging.

This work supports conclusions from previous studies that the choice of tools and databases used can have a large effect on findings [59,71], as evidenced in the differing relative abundances of *S. enterica* in individual samples between methods and the serovar and AMR prediction results. Differences in *S. enterica* relative abundances inferred with Centrifuge compared to Kraken2 and Bracken can be a result of differences in tool algorithms, parameters and databases used; in particular, Bracken analysis involves abundance re-estimation at a given taxonomic level, which was not done in the Centrifuge approach. It is also important to reiterate that taxonomic classifiers used to infer the relative abundances of taxa in the current study have been associated with false positive results due to low specificity [71], and thus, low-level detection of *S. enterica* reads could indicate false positive results, again highlighting that further work is necessary to determine the diagnostic potential of metagenomics. Whilst not so much of an issue for the case samples, which were cultured for the pathogen, the identification of *S. enterica* reads in the controls does not necessarily mean all of the healthy individuals are carriers. Use of more conservative approaches, for example, those using marker genes to aid classification, could potentially help to distinguish cases and controls and thus facilitate the establishment of diagnostic thresholds. Additional classification with MetaPhlAn4 indeed indicated the absence of *S. enterica* in the healthy control cohort, though the pathogen was also not identified in 12 of the culture-confirmed case samples; therefore, the use of more conservative methods may control potential false positive detection, at the risk of instead increasing false negative results. Additionally, *S. enterica* serovar Meleagridis was not identified in the sample from which this serovar was isolated through alignment and local assembly, as a high-quality reference genome was not available in our database.

Another important limitation noted by other authors is microbial coverage reduction due to host DNA presence, which can be particularly high in gastroenteritis cases, especially if these feature bloody diarrhoea [69,72]. There was no correlation between the relative abundance of NTS or NTS serovars and total metagenome reads in the current study, indicating that the pathogen detection signal was not linked to the overall sequencing depth. However, there was a large range in the total size of case metagenomes after host-depletion compared to the controls, which highlights the need for effective host DNA depletion methods prior to sequencing. This would allow increased sequencing depth of bacterial reads and facilitate more robust detection, for example, through the assembly of high-quality MAGs.

In conclusion, this study demonstrates the potential of metagenomics for direct pathogen identification from stool metagenomes, although the stage of infection and possible coinfections can affect diagnosis in the absence of the isolated pathogens. Reliable characterisation of the causative pathogen with metagenomics at the serovar and AMR genotype level may not be possible with the current methods and tools, especially when the resolution is reduced by high proportions of host reads in disease cases. This limits the suitability for epidemiological studies and guiding antimicrobial treatment. Improvements to the method could allow more precise pathogen characterisation for clinical use. This could include implementing long-read and adaptive sequencing [73] or hybrid-capture target enrichment [74] to increase resolution of the pathogen of interest and improvements in host depletion prior to sequencing. Improvements in analysis pipelines, for example using comprehensive databases with pathogen-specific markers [69], or tools able to discriminate between closely related organisms to reduce false positive hits, could also be beneficial.

## Supplementary material

10.1099/mgen.0.001547 Uncited Supplementary Material 1.

10.1099/mgen.0.001547 Uncited Supplementary Material 2.

## Abbreviations

AMR: antimicrobial resistance
CARD: Comprehensive Antibiotic Resistance Database
cgMLST: core genome multilocus sequence typing
ENA: European Nucleotide Archive
LMICs: low- and middle-income countries
MAG: metagenome-assembled genome
MDR: multidrug resistance
NCBI: National Center for Biotechnology Information
NMDS: non-metric multidimensional scaling
NTS: nontyphoidal *Salmonella*
OTU: operational taxonomic unit
SISTR: *Salmonella in silico* typing resource
STs: sequence types

## References

[1] Brenner FW, Villar RG, Angulo FJ, Tauxe R, Swaminathan B (2000). *Salmonella* nomenclature. J Clin Microbiol.

[2] Ryan MP, O’Dwyer J, Adley CC (2017). Evaluation of the complex nomenclature of the clinically and veterinary significant pathogen *Salmonella.*. Biomed Res Int.

[3] Ehuwa O, Jaiswal AK, Jaiswal S (2021). *Salmonella*, food safety and food handling practices. Foods.

[4] Gal-Mor O, Boyle EC, Grassl GA (2014). Same species, different diseases: how and why typhoidal and non-typhoidal *Salmonella enterica* serovars differ. Front Microbiol.

[5] Ugboko HU, Nwinyi OC, Oranusi SU, Oyewale JO (2020). Childhood diarrhoeal diseases in developing countries. Heliyon.

[6] Thompson CN, Phan VTM, Le TPT, Pham TNT, Hoang LP (2013). Epidemiological features and risk factors of *Salmonella* gastroenteritis in children resident in Ho Chi Minh City, Vietnam. *Epidemiol Infect*.

[7] Duong VT, The HC, Nhu TDH, Tuyen HT, Campbell JI (2020). Genomic serotyping, clinical manifestations, and antimicrobial resistance of nontyphoidal *Salmonella* gastroenteritis in hospitalized children in Ho Chi Minh City, Vietnam. *J Clin Microbiol*.

[8] Kinh N (2010). Situation analysis: antibiotic use and resistance in Vietnam. CDDEP.

[9] Van TTH, Nguyen HNK, Smooker PM, Coloe PJ (2012). The antibiotic resistance characteristics of non-typhoidal *Salmonella enterica* isolated from food-producing animals, retail meat and humans in South East Asia. Int J Food Microbiol.

[10] Gilligan PH, Rosenberg E, DeLong EF, Lory S, Stackebrandt E, Thompson F (2013). The Prokaryotes: Human Microbiology.

[11] Uelze L, Grützke J, Borowiak M, Hammerl JA, Juraschek K (2020). Typing methods based on whole genome sequencing data. *One Health Outlook*.

[12] Aljahdali NH, Sanad YM, Han J, Foley SL (2020). Current knowledge and perspectives of potential impacts of *Salmonella enterica* on the profile of the gut microbiota. BMC Microbiol.

[13] Afgan E, Baker D, Batut B, van den Beek M, Bouvier D (2018). The galaxy platform for accessible, reproducible and collaborative biomedical analyses: 2018 update. Nucleic Acids Res.

[14] Connor TR, Loman NJ, Thompson S, Smith A, Southgate J (2016). CLIMB (the cloud infrastructure for microbial bioinformatics): an online resource for the medical microbiology community. Microb Genom.

[15] Chen S, Zhou Y, Chen Y, Gu J (2018). fastp: an ultra-fast all-in-one FASTQ preprocessor. Bioinformatics.

[16] Prjibelski A, Antipov D, Meleshko D, Lapidus A, Korobeynikov A (2020). Using SPAdes de novo assembler. Curr Protoc Bioinformatics.

[17] Gurevich A, Saveliev V, Vyahhi N, Tesler G (2013). QUAST: quality assessment tool for genome assemblies. Bioinformatics.

[18] Parks DH, Imelfort M, Skennerton CT, Hugenholtz P, Tyson GW (2015). CheckM: assessing the quality of microbial genomes recovered from isolates, single cells, and metagenomes. Genome Res.

[19] Li H (2013). Aligning sequence reads, clone sequences and assembly contigs with BWA-MEM. arXiv.

[20] Li H, Durbin R (2009). Fast and accurate short read alignment with Burrows-Wheeler transform. Bioinformatics.

[21] Seemann T (2014). Prokka: rapid prokaryotic genome annotation. Bioinformatics.

[22] Page AJ, Cummins CA, Hunt M, Wong VK, Reuter S (2015). Roary: rapid large-scale prokaryote pan genome analysis. Bioinformatics.

[23] Trifinopoulos J, Nguyen L-T, von Haeseler A, Minh BQ (2016). W-IQ-TREE: a fast online phylogenetic tool for maximum likelihood analysis. Nucleic Acids Res.

[24] Hoang DT, Chernomor O, von Haeseler A, Minh BQ, Vinh LS (2018). UFBoot2: improving the ultrafast bootstrap approximation. Mol Biol Evol.

[25] Guindon S, Dufayard J-F, Lefort V, Anisimova M, Hordijk W (2010). New algorithms and methods to estimate maximum-likelihood phylogenies: assessing the performance of PhyML 3.0. Syst Biol.

[26] Yoshida CE, Kruczkiewicz P, Laing CR, Lingohr EJ, Gannon VPJ (2016). The *Salmonella in silico* typing resource (SISTR): an open web-accessible tool for rapidly typing and subtyping draft *Salmonella* genome assemblies. PLoS ONE.

[27] Clausen P, Aarestrup FM, Lund O (2018). Rapid and precise alignment of raw reads against redundant databases with KMA. BMC Bioinformatics.

[28] Zankari E, Hasman H, Cosentino S, Vestergaard M, Rasmussen S (2012). Identification of acquired antimicrobial resistance genes. J Antimicrob Chemother.

[29] Magnet S, Courvalin P, Lambert T (1999). Activation of the cryptic *aac(6’)-Iy* aminoglycoside resistance gene of *Salmonella* by a chromosomal deletion generating a transcriptional fusion. J Bacteriol.

[30] Zankari E, Allesøe R, Joensen KG, Cavaco LM, Lund O (2017). PointFinder: a novel web tool for WGS-based detection of antimicrobial resistance associated with chromosomal point mutations in bacterial pathogens. J Antimicrob Chemother.

[31] Pereira-Dias J, Nguyen Ngoc Minh C, Tran Thi Hong C, Nguyen Thi Nguyen T, Ha Thanh T (2021). The gut microbiome of healthy vietnamese adults and children is a major reservoir for resistance genes against critical antimicrobials. J Infect Dis.

[32] Constantinides B, Hunt M, Crook DW (2023). Hostile: accurate decontamination of microbial host sequences. Bioinformatics.

[33] Wood DE, Salzberg SL (2014). Kraken: ultrafast metagenomic sequence classification using exact alignments. Genome Biol.

[34] Lu J, Breitwieser FP, Thielen P, Salzberg SL (2017). Bracken: estimating species abundance in metagenomics data. PeerJ Comput Sci.

[35] Kim D, Song L, Breitwieser FP, Salzberg SL (2016). Centrifuge: rapid and sensitive classification of metagenomic sequences. Genome Res.

[36] Blanco-Míguez A, Beghini F, Cumbo F, McIver LJ, Thompson KN (2023). Extending and improving metagenomic taxonomic profiling with uncharacterized species using MetaPhlAn 4. Nat Biotechnol.

[37] R Core Team (2021). https://www.R-project.org/.

[38] Oksanen J, Simpson G, Blanchet F, Kindt R, Legendre P (2022). Vegan: community ecology package. https://CRAN.R-project.org/package=vegan.

[39] Marçais G, Delcher AL, Phillippy AM, Coston R, Salzberg SL (2018). MUMmer4: a fast and versatile genome alignment system. PLoS Comput Biol.

[40] Li D, Liu C-M, Luo R, Sadakane K, Lam T-W (2015). MEGAHIT: an ultra-fast single-node solution for large and complex metagenomics assembly via succinct de bruijn graph. Bioinformatics.

[41] Langmead B, Salzberg SL (2012). Fast gapped-read alignment with bowtie 2. Nat Methods.

[42] Kang DD, Li F, Kirton E, Thomas A, Egan R (2019). MetaBAT 2: an adaptive binning algorithm for robust and efficient genome reconstruction from metagenome assemblies. PeerJ.

[43] Wu Y-W, Simmons BA, Singer SW (2016). MaxBin 2.0: an automated binning algorithm to recover genomes from multiple metagenomic datasets. Bioinformatics.

[44] von Meijenfeldt FAB, Arkhipova K, Cambuy DD, Coutinho FH, Dutilh BE (2019). Robust taxonomic classification of uncharted microbial sequences and bins with CAT and BAT. Genome Biol.

[45] Khachatryan L, de Leeuw RH, Kraakman MEM, Pappas N, Te Raa M (2020). Taxonomic classification and abundance estimation using 16S and WGS-A comparison using controlled reference samples. Forensic Sci Int Genet.

[46] Hasman H, Saputra D, Sicheritz-Ponten T, Lund O, Svendsen CA (2014). Rapid whole-genome sequencing for detection and characterization of microorganisms directly from clinical samples. J Clin Microbiol.

[47] Alcock BP, Huynh W, Chalil R, Smith KW, Raphenya AR (2023). CARD 2023: expanded curation, support for machine learning, and resistome prediction at the comprehensive antibiotic resistance database. Nucleic Acids Res.

[48] Hussein NH, Al-Kadmy IMS, Taha BM, Hussein JD (2021). Mobilized colistin resistance (mcr) genes from 1 to 10: a comprehensive review. Mol Biol Rep.

[49] Zhang Z, Schwartz S, Wagner L, Miller W (2000). A greedy algorithm for aligning DNA sequences. J Comput Biol.

[50] Morgulis A, Coulouris G, Raytselis Y, Madden TL, Agarwala R (2008). Database indexing for production MegaBLAST searches. Bioinformatics.

[51] Thompson CN, Phan MVT, Hoang NVM, Minh PV, Vinh NT (2015). A prospective multi-center observational study of children hospitalized with diarrhea in Ho Chi Minh City, Vietnam. *Am J Trop Med Hyg*.

[52] Navidad JF, Griswold DJ, Gradus MS, Bhattacharyya S (2013). Evaluation of luminex xTAG gastrointestinal pathogen analyte-specific reagents for high-throughput, simultaneous detection of bacteria, viruses, and parasites of clinical and public health importance. J Clin Microbiol.

[53] Deng J, Luo X, Wang R, Jiang L, Ding X (2015). A comparison of luminex xTAG® gastrointestinal pathogen panel (xTAG GPP) and routine tests for the detection of enteropathogens circulating in Southern China. Diagn Microbiol Infect Dis.

[54] Duong VT, Phat VV, Tuyen HT, Dung TTN, Trung PD (2016). Evaluation of luminex xTAG gastrointestinal pathogen panel assay for detection of multiple diarrheal pathogens in fecal samples in Vietnam. J Clin Microbiol.

[55] Gal-Mor O (2019). Persistent infection and long-term carriage of typhoidal and nontyphoidal Salmonellae. Clin Microbiol Rev.

[56] Chaudhuri RR, Henderson IR (2012). The evolution of the *Escherichia coli* phylogeny. Infect Genet Evol.

[57] Crump JA, Sjölund-Karlsson M, Gordon MA, Parry CM (2015). Epidemiology, clinical presentation, laboratory diagnosis, antimicrobial resistance, and antimicrobial management of invasive *Salmonella* infections. Clin Microbiol Rev.

[58] Sanguineti D, Zampieri G, Treu L, Campanaro S (2024). Metapresence: a tool for accurate species detection in metagenomics based on the genome-wide distribution of mapping reads. mSystems.

[59] Zhou Y, Wylie KM, El Feghaly RE, Mihindukulasuriya KA, Elward A (2016). Metagenomic approach for identification of the pathogens associated with diarrhea in stool specimens. J Clin Microbiol.

[60] Meziti A, Rodriguez-R LM, Hatt JK, Peña-Gonzalez A, Levy K (2021). The reliability of metagenome-assembled genomes (MAGs) in representing natural populations: insights from comparing MAGs against isolate genomes derived from the same fecal sample. Appl Environ Microbiol.

[61] Bowers RM, Kyrpides NC, Stepanauskas R, Harmon-Smith M, Doud D (2017). Minimum information about a single amplified genome (MISAG) and a metagenome-assembled genome (MIMAG) of bacteria and archaea. Nat Biotechnol.

[62] Morbidity and mortality weekly report (MMWR) (2010). Multiple-serotype *Salmonella* gastroenteritis outbreak after a reception --- connecticut, 2009. Centers for Disease Control and Prevention.

[63] Gicquelais RE, Morris JF, Matthews HS, Gladden L, Safi H (2012). Multiple-serotype *Salmonella* outbreaks in two state prisons — arkansas, august 2012. MMWR Morb Mortal Wkly Rep.

[64] Rudder SJ, Djeghout B, Elumogo N, Janecko N, Langridge GC (2025). Genomic diversity of non-typhoidal *Salmonella* found in patients suffering from gastroenteritis in Norfolk, UK. Microb Genom.

[65] The HC, Le S-N (2022). Dynamic of the human gut microbiome under infectious diarrhea. Curr Opin Microbiol.

[66] Liu H, Guo M, Jiang Y, Cao Y, Qian Q (2019). Diagnosing and tracing the pathogens of infantile infectious diarrhea by amplicon sequencing. Gut Pathog.

[67] Sibinelli-Sousa S, de Araújo-Silva AL, Hespanhol JT, Bayer-Santos E (2022). Revisiting the steps of *Salmonella* gut infection with a focus on antagonistic interbacterial interactions. FEBS J.

[68] The HC, Florez de Sessions P, Jie S, Pham Thanh D, Thompson CN (2018). Assessing gut microbiota perturbations during the early phase of infectious diarrhea in vietnamese children. Gut Microbes.

[69] Peterson C-L, Alexander D, Chen JC-Y, Adam H, Walker M (2022). Clinical metagenomics is increasingly accurate and affordable to detect enteric bacterial pathogens in stool. Microorganisms.

[70] Pop M, Walker AW, Paulson J, Lindsay B, Antonio M (2014). Diarrhea in young children from low-income countries leads to large-scale alterations in intestinal microbiota composition. Genome Biol.

[71] Doster E, Rovira P, Noyes NR, Burgess BA, Yang X (2019). A cautionary report for pathogen identification using shotgun metagenomics; a comparison to aerobic culture and polymerase chain reaction for *Salmonella enterica* identification. Front Microbiol.

[72] Huang AD, Luo C, Pena-Gonzalez A, Weigand MR, Tarr CL (2017). Metagenomics of two severe foodborne outbreaks provides diagnostic signatures and signs of coinfection not attainable by traditional methods. Appl Environ Microbiol.

[73] Martin S, Heavens D, Lan Y, Horsfield S, Clark MD (2022). Nanopore adaptive sampling: a tool for enrichment of low abundance species in metagenomic samples. Genome Biol.

[74] Quek ZBR, Ng SH (2024). Hybrid-capture target enrichment in human pathogens: identification, evolution, biosurveillance, and genomic epidemiology. Pathogens.

