# Supplementary material for: Metagenomic identification of disease-causing Salmonella enterica serovars and antimicrobial resistance genes from paediatric faecal samples
Source: Microb Genom. 2025 Oct 27;11(10):001547. doi: 10.1099/mgen.0.001547 (PMC12558408; doi:10.1099/mgen.0.001547)
Supplement: Uncited Supplementary Material 1. [file mgen-11-01547-s001.pdf]

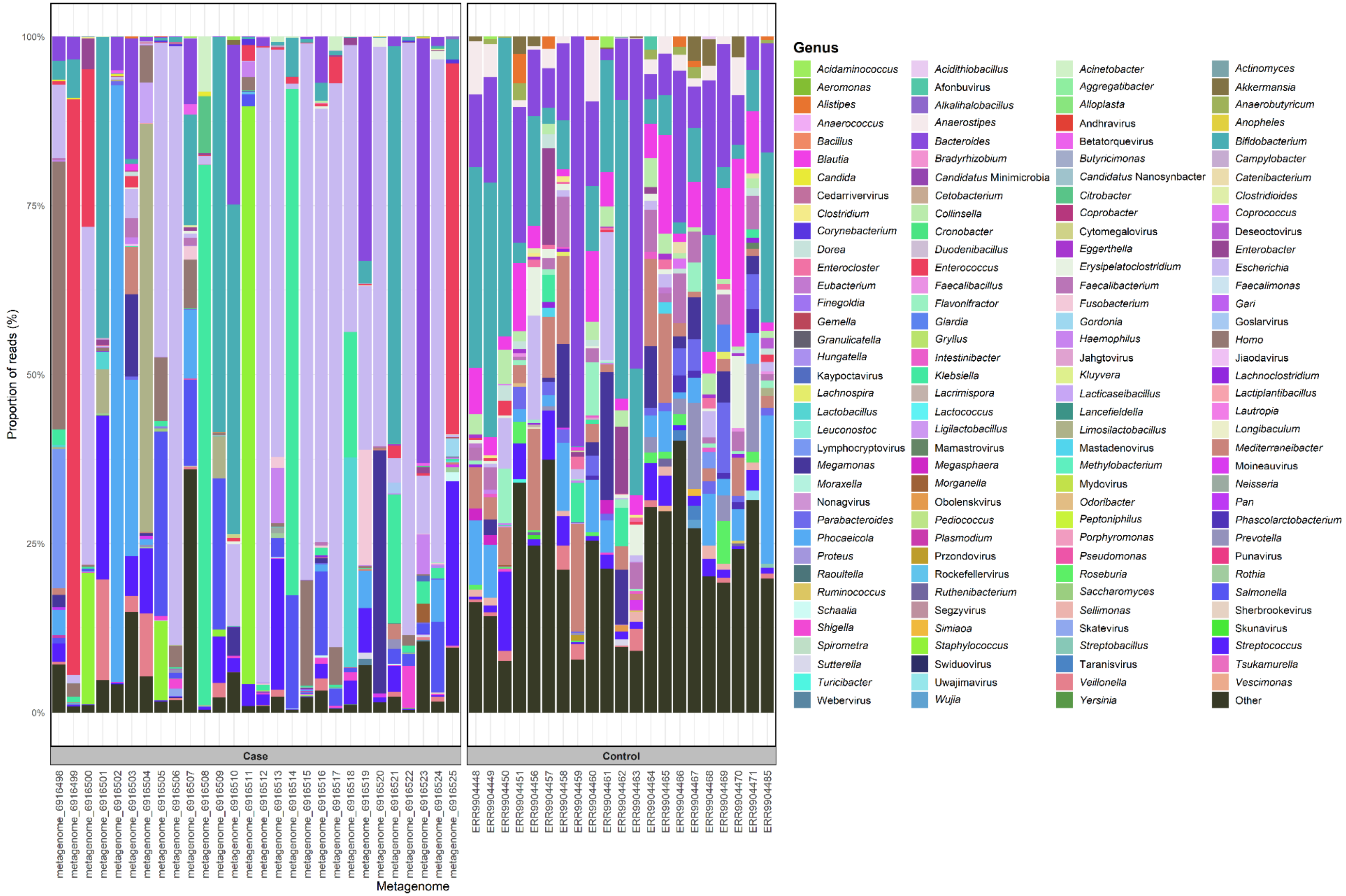

Figure S1: The 20 most abundant genera based on Bracken results across the case and control metagenomes, with remaining classifications grouped into the 'other' category in each sample

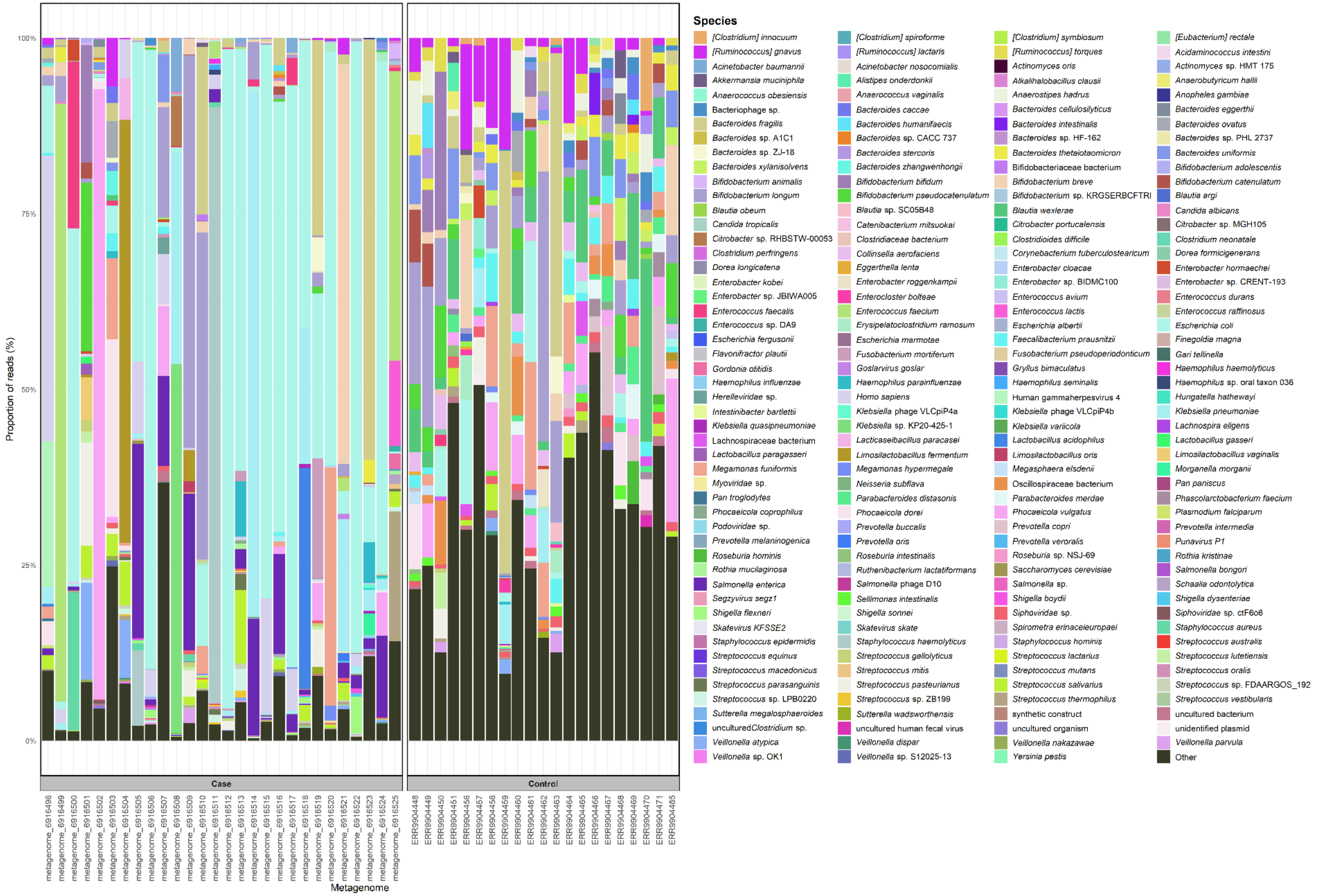

Figure S2: The 20 most abundant species based on Bracken results across the case and control metagenomes, with remaining classifications grouped into the 'other' category in each sample

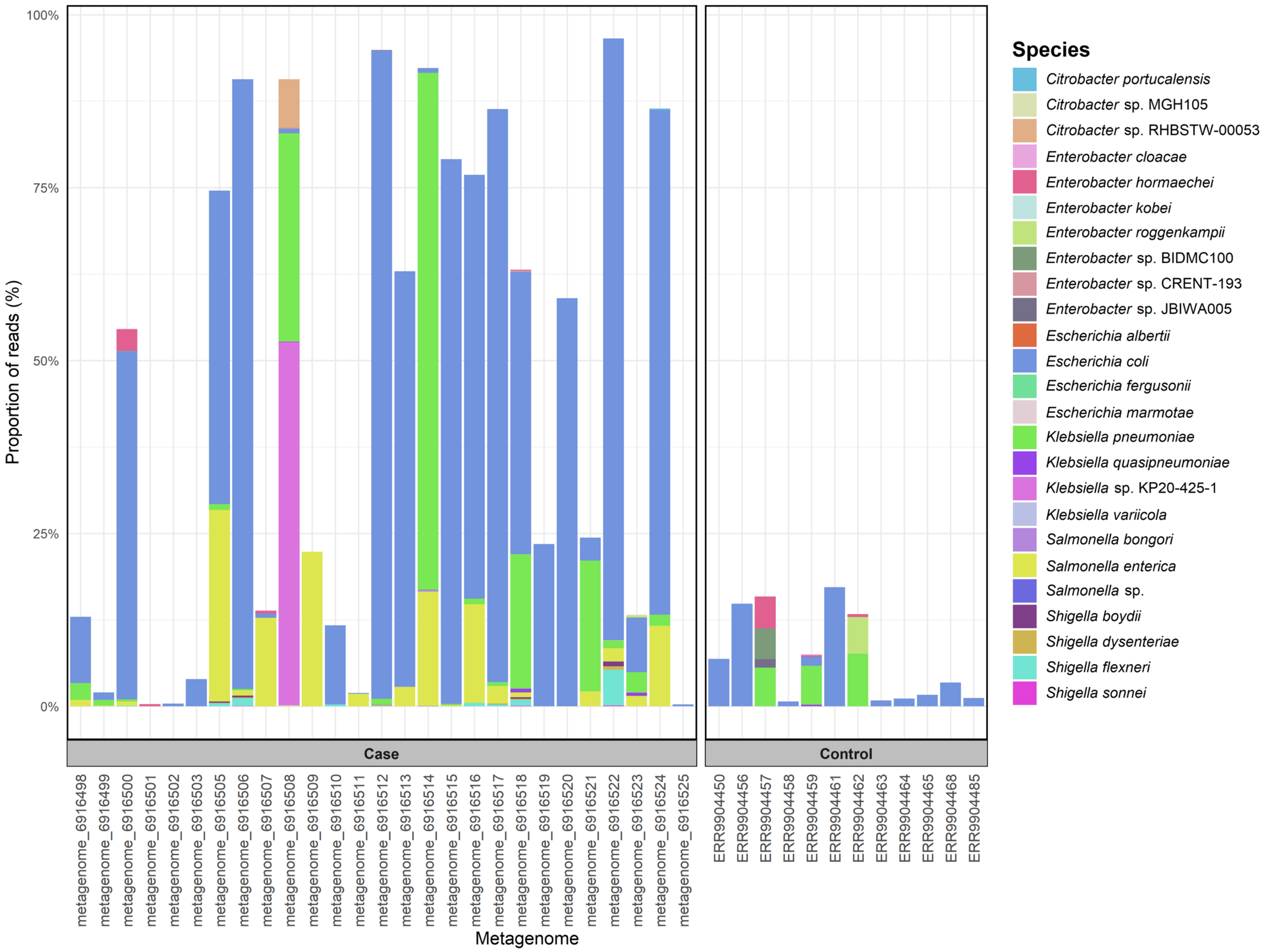

Figure S3: Enterobacteriaceae relative abundance across the case and control metagenomes, based on the top 20 Bracken species in each metagenome

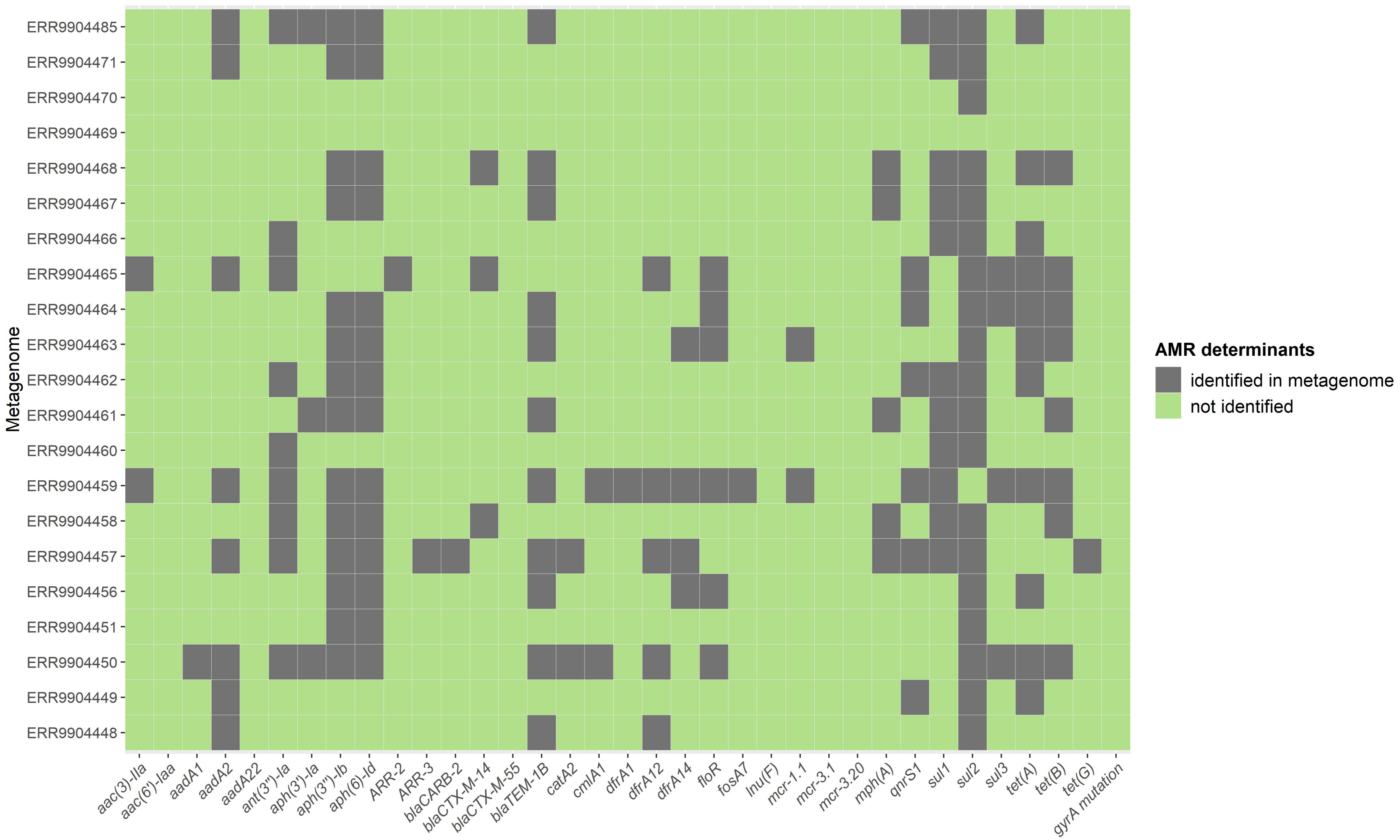

Figure S4: Identification of antimicrobial resistance (AMR) genes associated with the *S. enterica* genomes in the control faecal metagenomes
